# Supplementary figures and images for: MiR-27a inhibits the growth and metastasis of multiple myeloma through regulating Th17/Treg balance
Source: PLoS One. 2024 Oct 16;19(10):e0311419. doi: 10.1371/journal.pone.0311419 (PMC11482689; doi:10.1371/journal.pone.0311419)

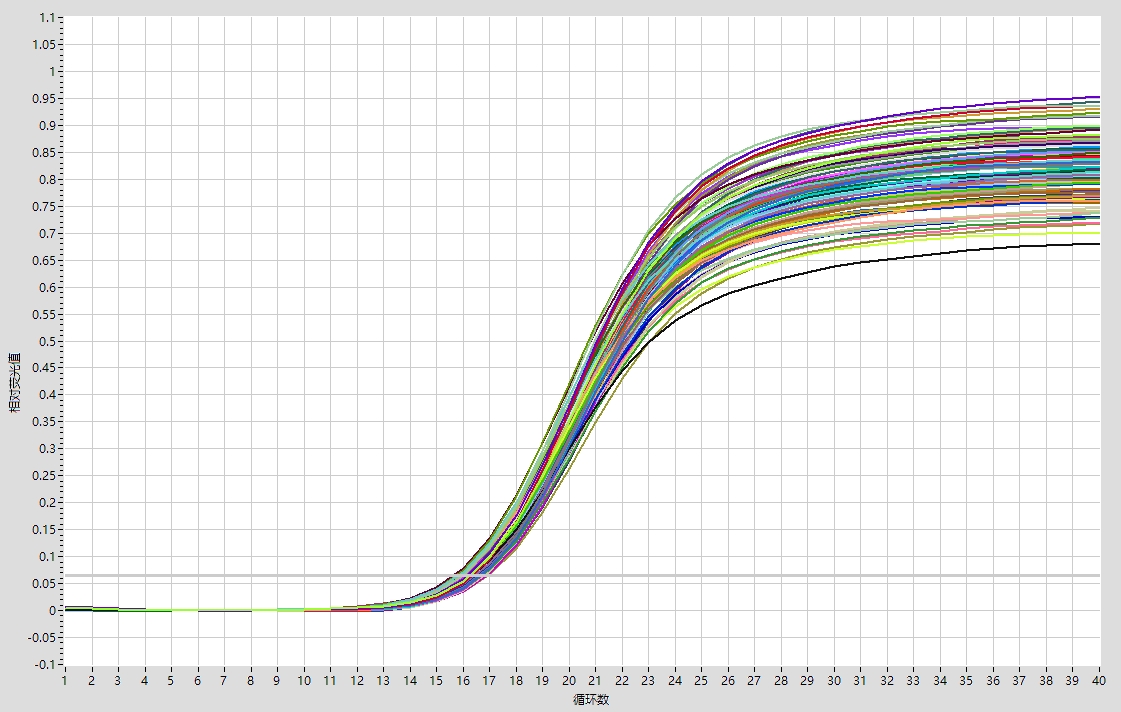

Supplement: S1 File — (ZIP) [file pone.0311419.s001.zip › PCR/micRNA-27a└⌐╘÷╟·╧▀.jpeg]

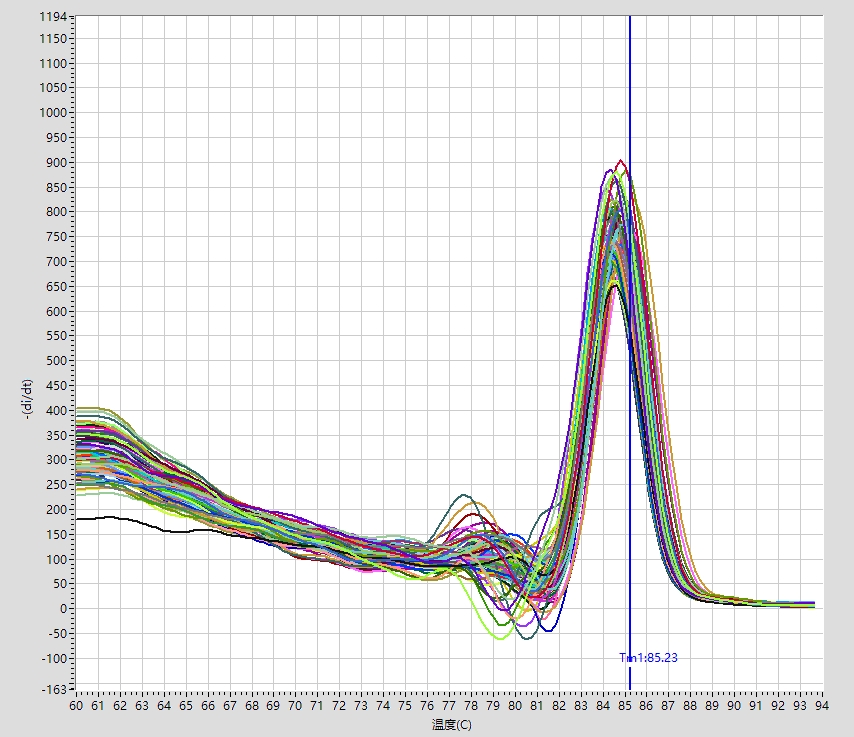

Supplement: S1 File — (ZIP) [file pone.0311419.s001.zip › PCR/micRNA-27a╚▄╜Γ╟·╧▀.jpeg]

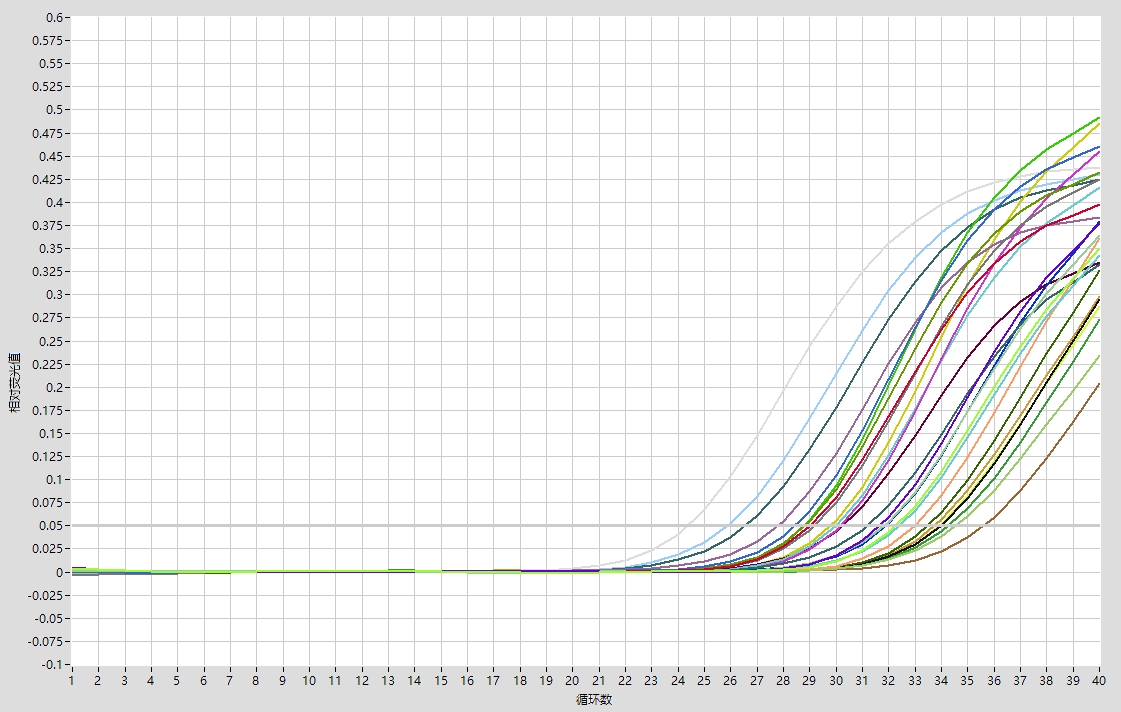

Supplement: S1 File — (ZIP) [file pone.0311419.s001.zip › PCR/U6└⌐╘÷╟·╧▀.jpeg]

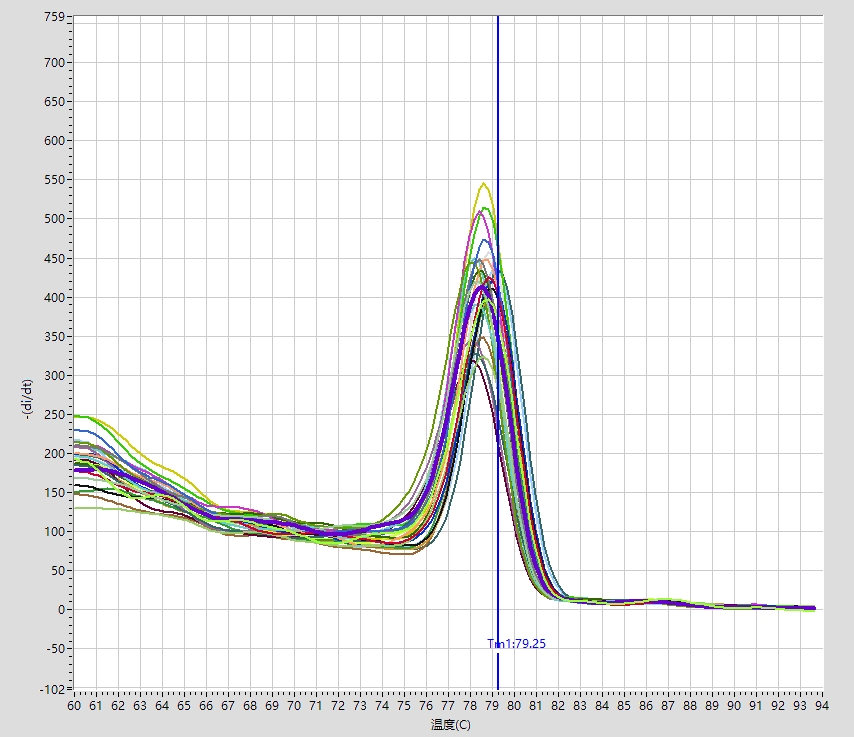

Supplement: S1 File — (ZIP) [file pone.0311419.s001.zip › PCR/U6╚█╜Γ╟·╧▀.jpeg]

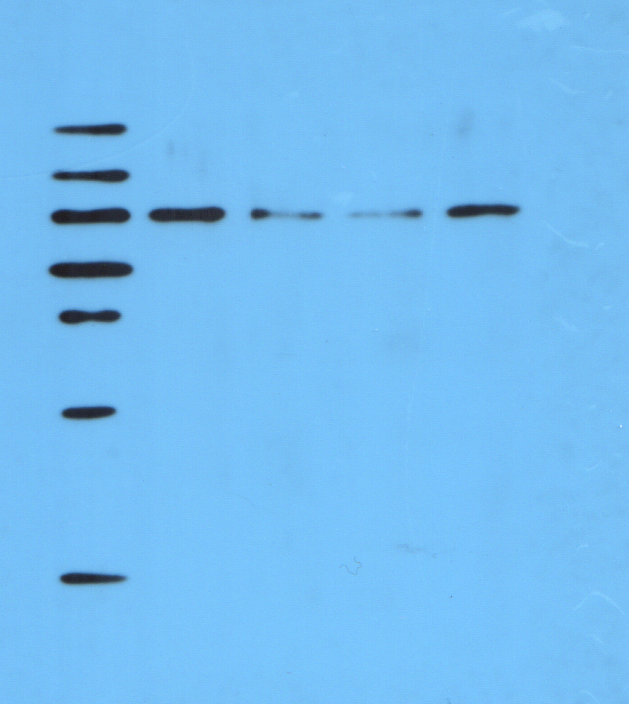

Supplement: S2 File — Detailed information on the antibody used, sample preparation, and exposure times is also provided. (ZIP) [file pone.0311419.s002.zip › wb/P-AKT.png]

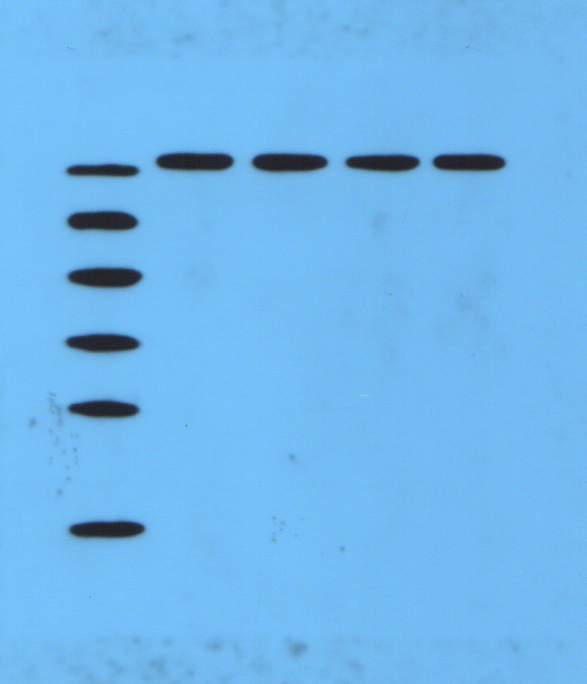

Supplement: S2 File — Detailed information on the antibody used, sample preparation, and exposure times is also provided. (ZIP) [file pone.0311419.s002.zip › wb/PI3K .png]

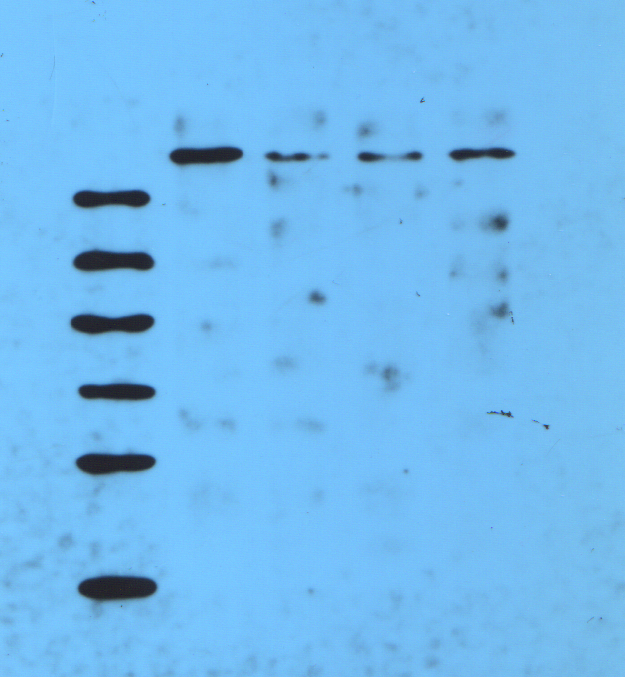

Supplement: S2 File — Detailed information on the antibody used, sample preparation, and exposure times is also provided. (ZIP) [file pone.0311419.s002.zip › wb/P-mTOR.png]

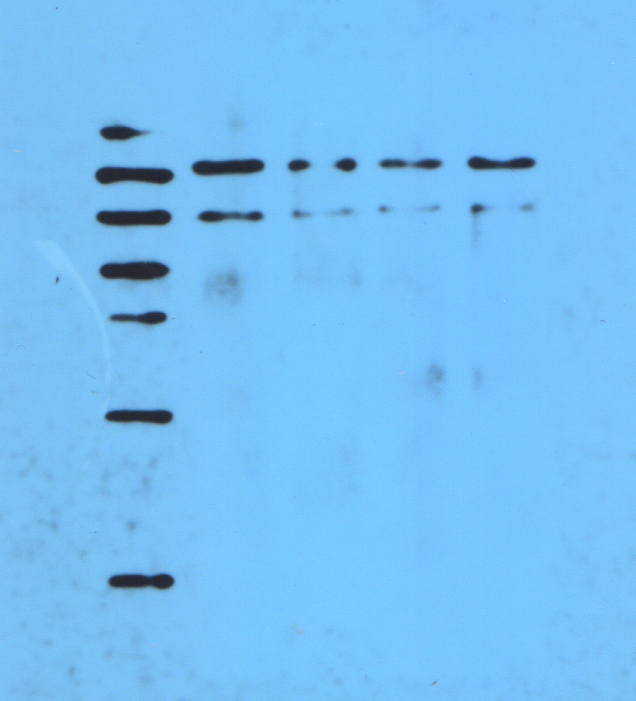

Supplement: S2 File — Detailed information on the antibody used, sample preparation, and exposure times is also provided. (ZIP) [file pone.0311419.s002.zip › wb/P-PI3K .png]

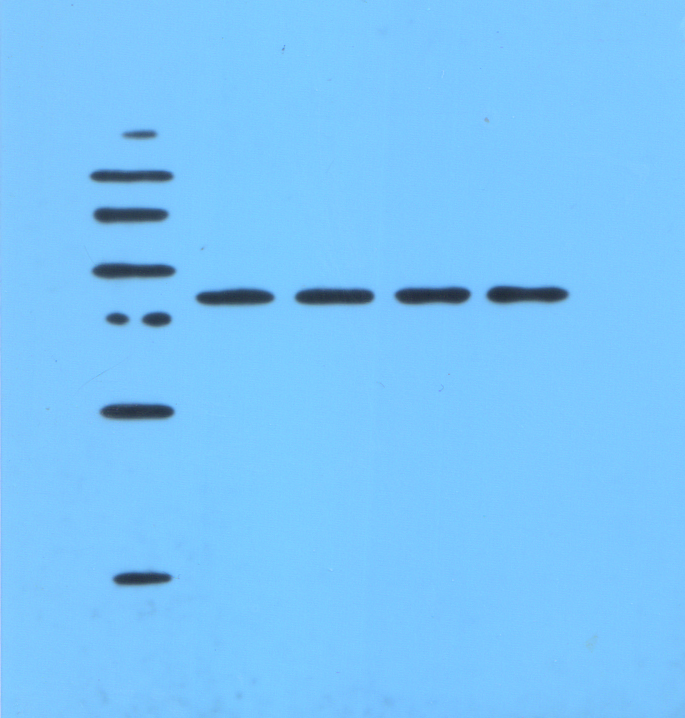

Supplement: S2 File — Detailed information on the antibody used, sample preparation, and exposure times is also provided. (ZIP) [file pone.0311419.s002.zip › wb/aó-Actin .png]

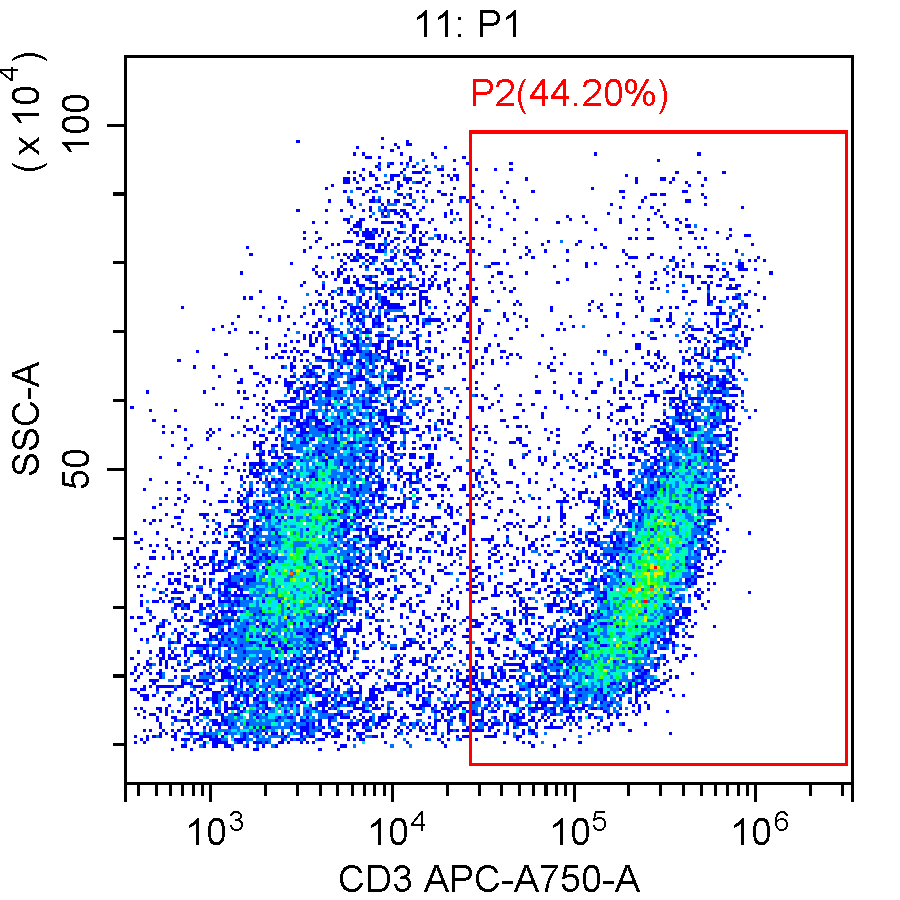

Supplement: S3 File — (ZIP) [file pone.0311419.s003.zip › ┴≈╩╜╝°╢¿+╡≥═÷/T╧╕░√▒╚╓╡/11.bmp]

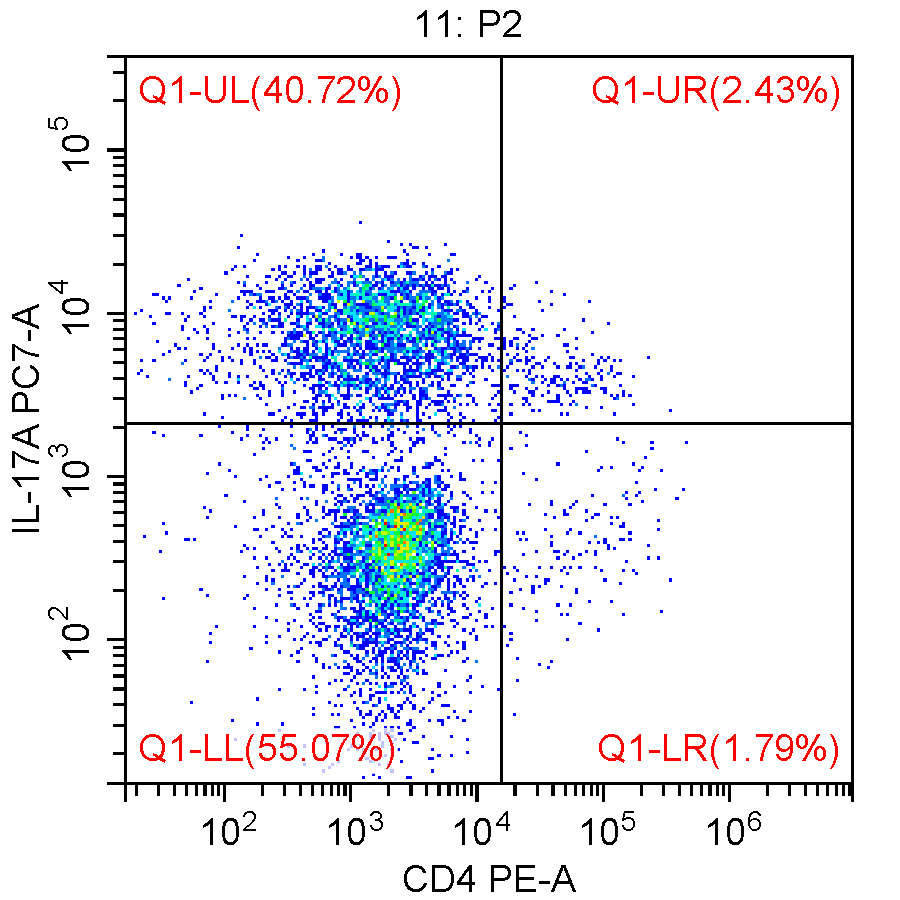

Supplement: S3 File — (ZIP) [file pone.0311419.s003.zip › ┴≈╩╜╝°╢¿+╡≥═÷/T╧╕░√▒╚╓╡/11-1.bmp]

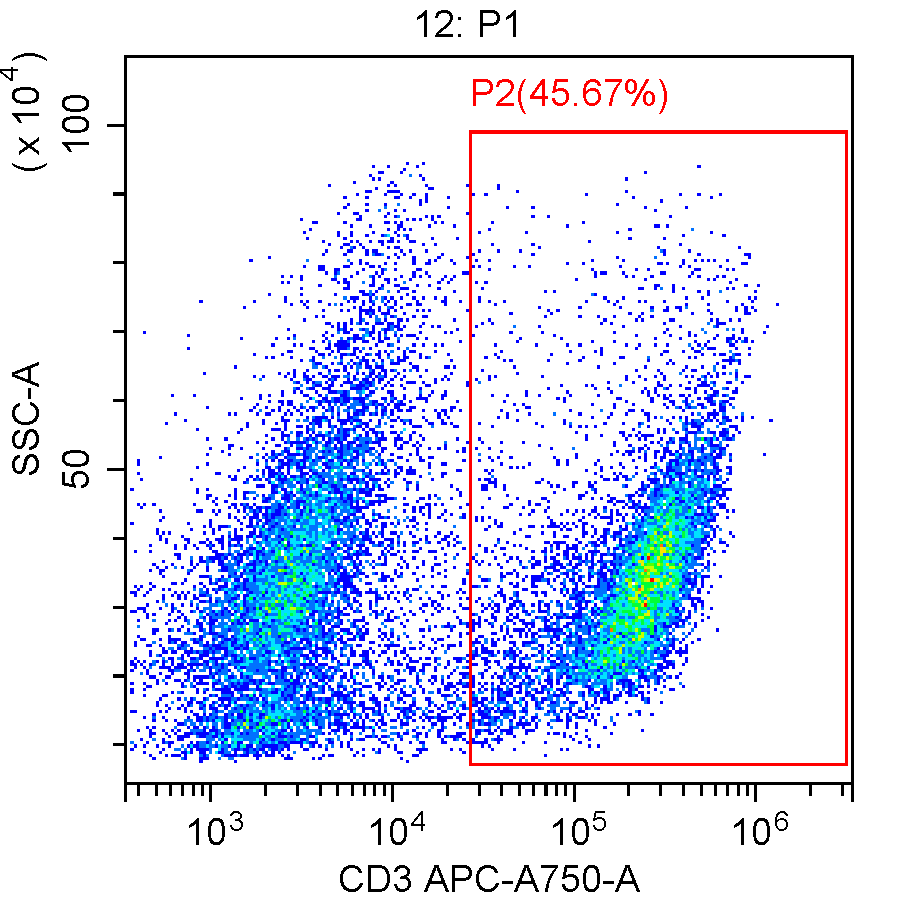

Supplement: S3 File — (ZIP) [file pone.0311419.s003.zip › ┴≈╩╜╝°╢¿+╡≥═÷/T╧╕░√▒╚╓╡/12.bmp]

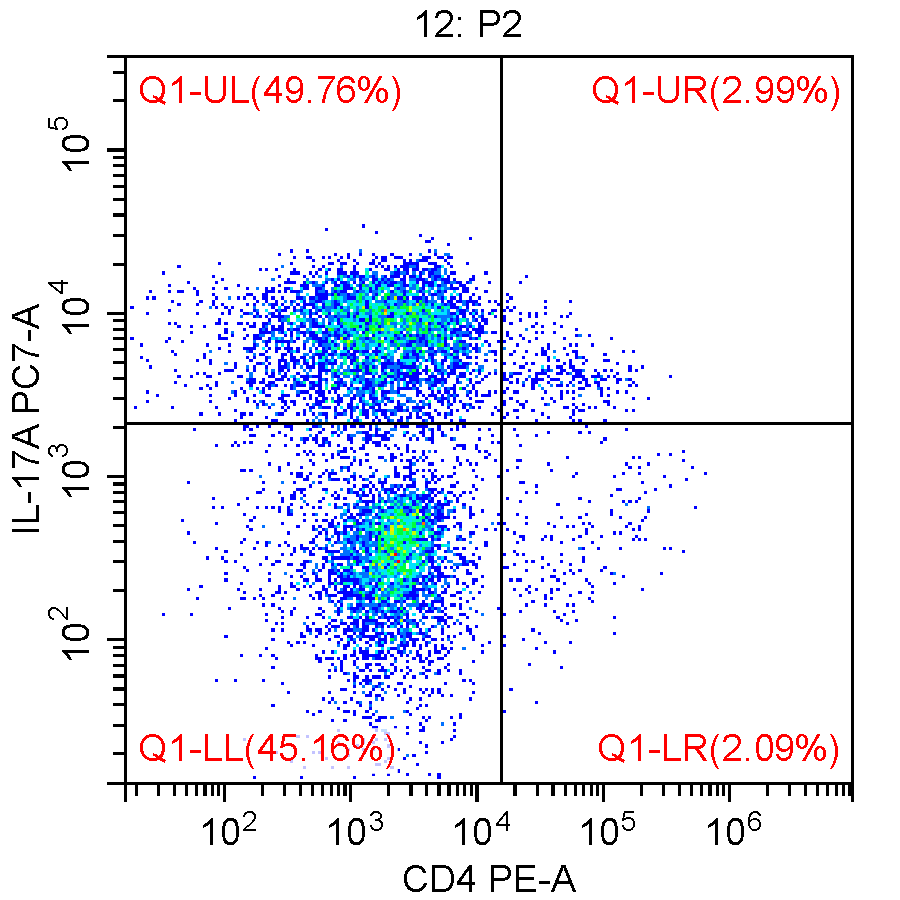

Supplement: S3 File — (ZIP) [file pone.0311419.s003.zip › ┴≈╩╜╝°╢¿+╡≥═÷/T╧╕░√▒╚╓╡/12-1.bmp]

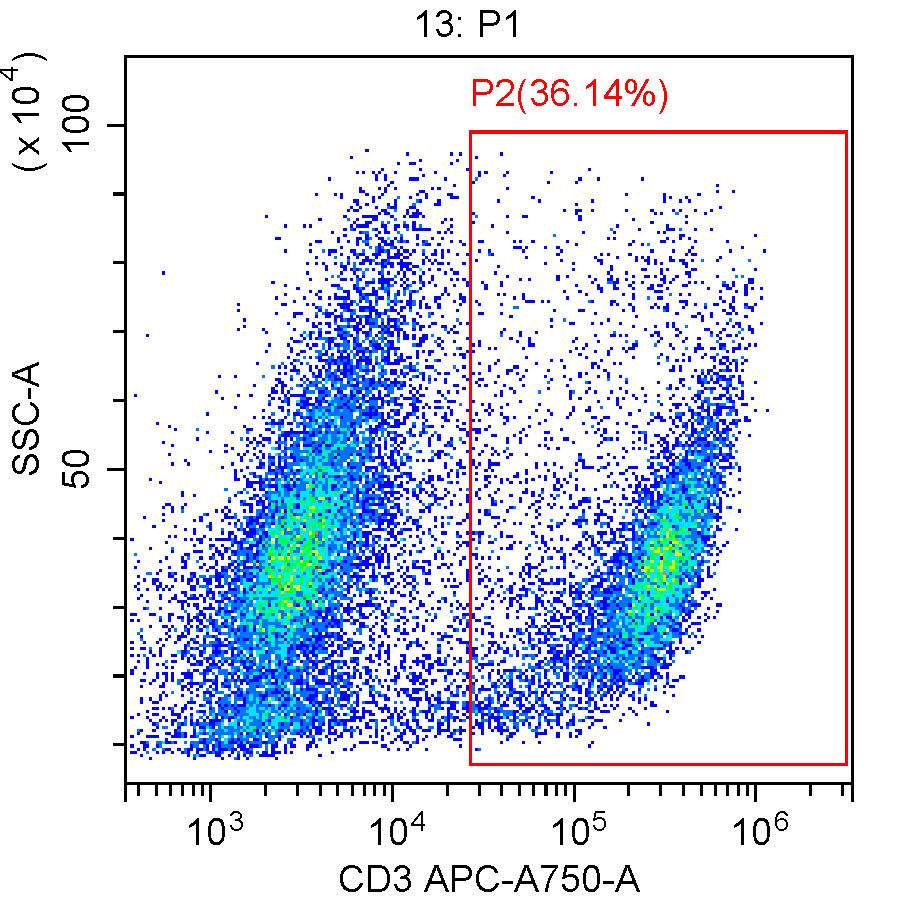

Supplement: S3 File — (ZIP) [file pone.0311419.s003.zip › ┴≈╩╜╝°╢¿+╡≥═÷/T╧╕░√▒╚╓╡/13.bmp]

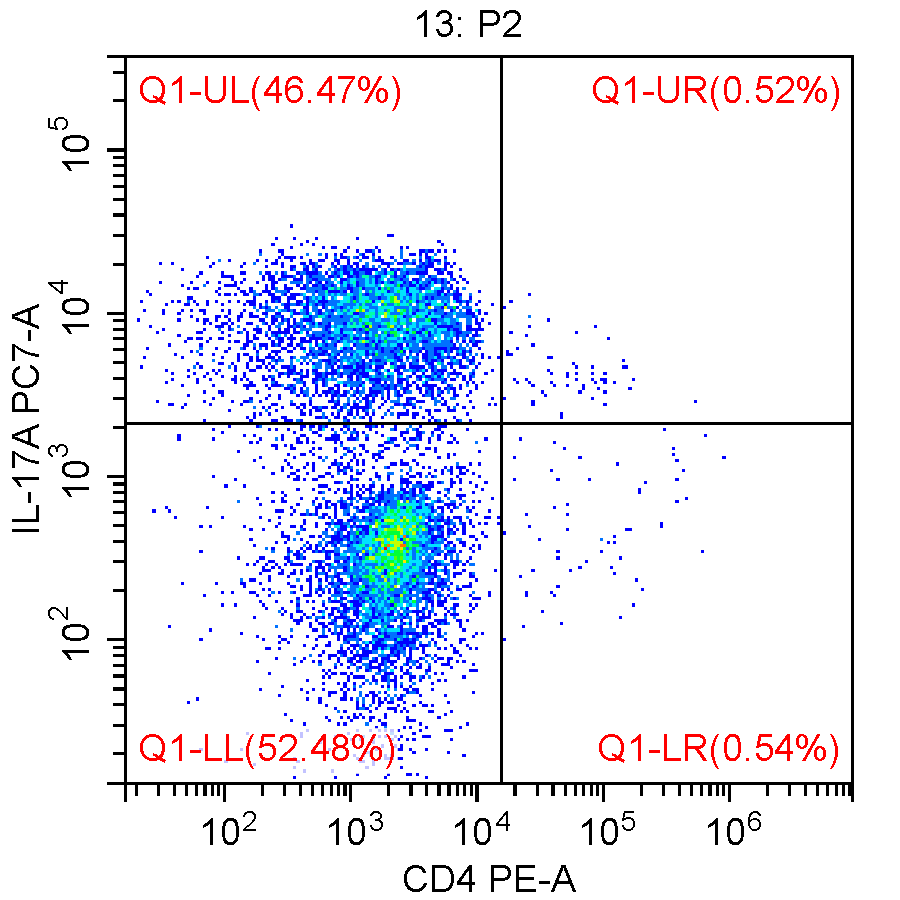

Supplement: S3 File — (ZIP) [file pone.0311419.s003.zip › ┴≈╩╜╝°╢¿+╡≥═÷/T╧╕░√▒╚╓╡/13-1.bmp]

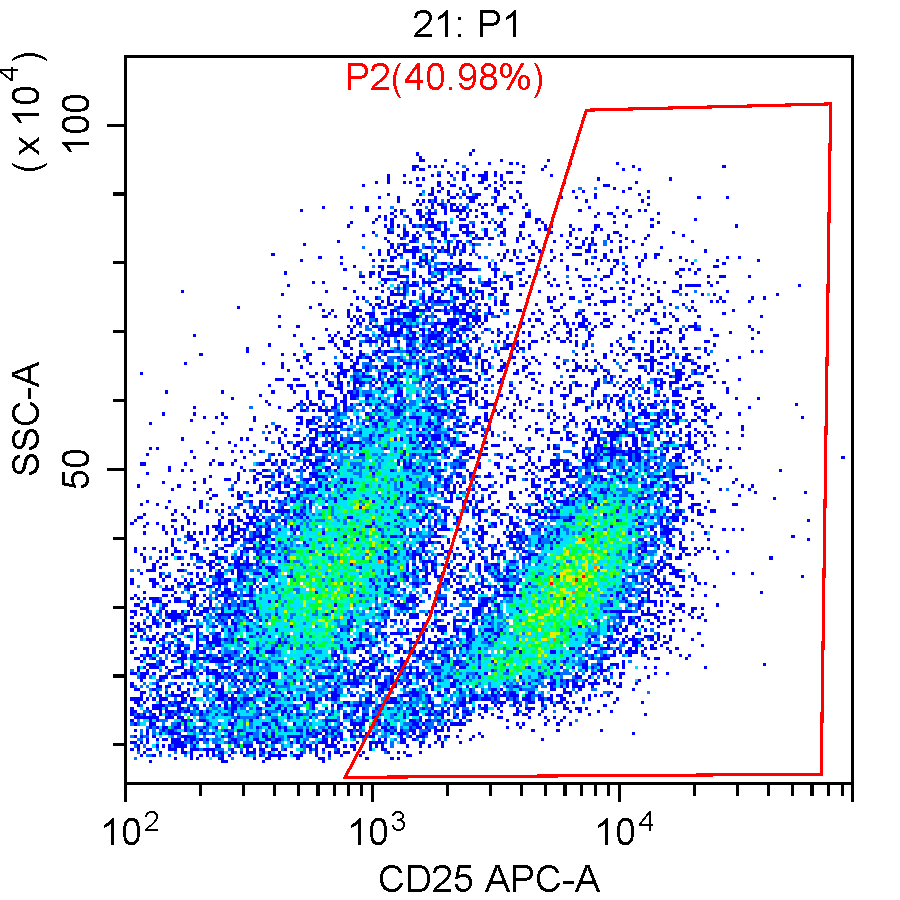

Supplement: S3 File — (ZIP) [file pone.0311419.s003.zip › ┴≈╩╜╝°╢¿+╡≥═÷/T╧╕░√▒╚╓╡/21.bmp]

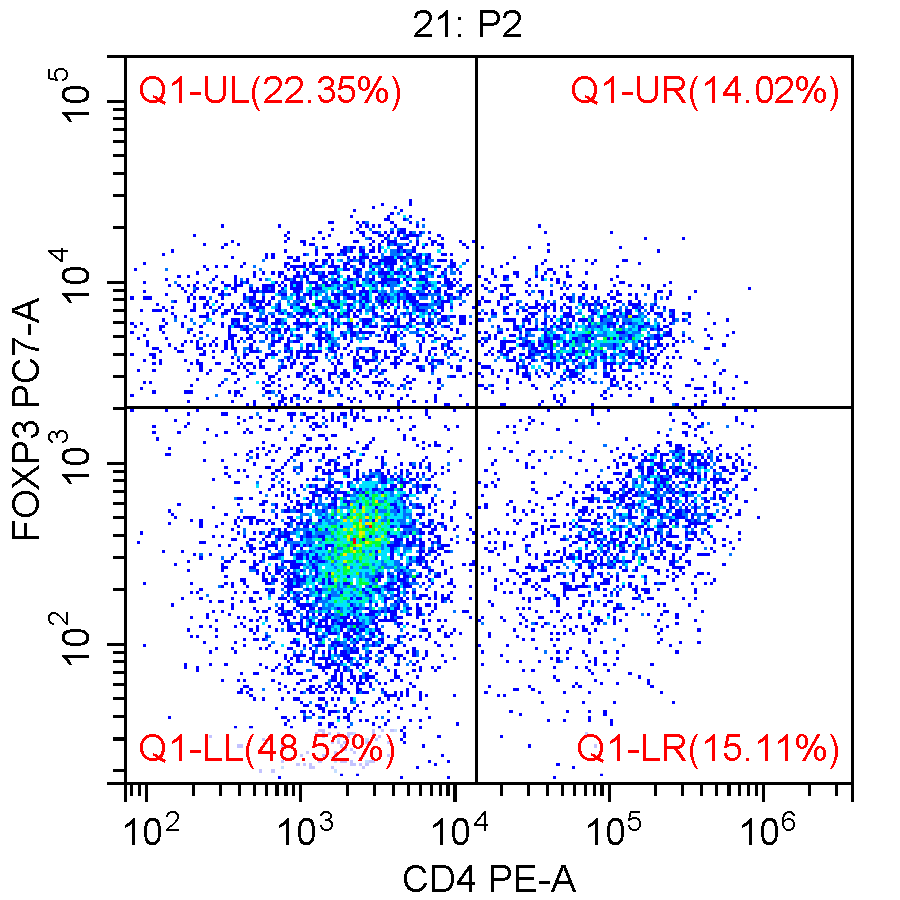

Supplement: S3 File — (ZIP) [file pone.0311419.s003.zip › ┴≈╩╜╝°╢¿+╡≥═÷/T╧╕░√▒╚╓╡/21-1.bmp]

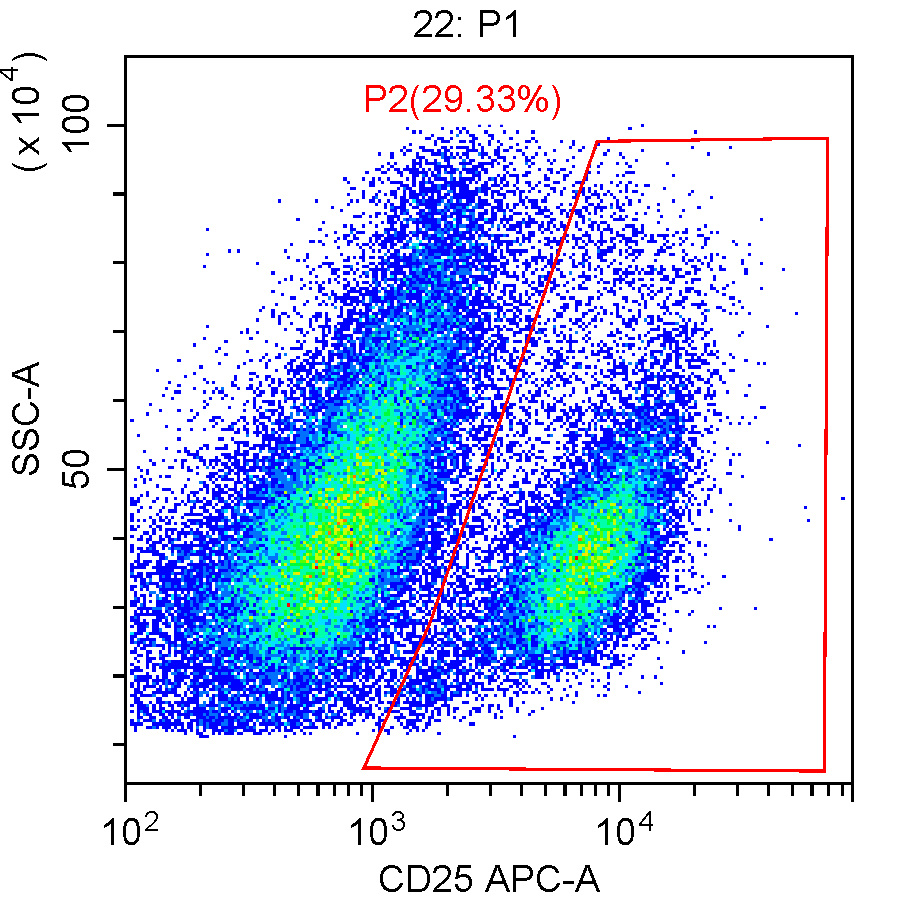

Supplement: S3 File — (ZIP) [file pone.0311419.s003.zip › ┴≈╩╜╝°╢¿+╡≥═÷/T╧╕░√▒╚╓╡/22.bmp]

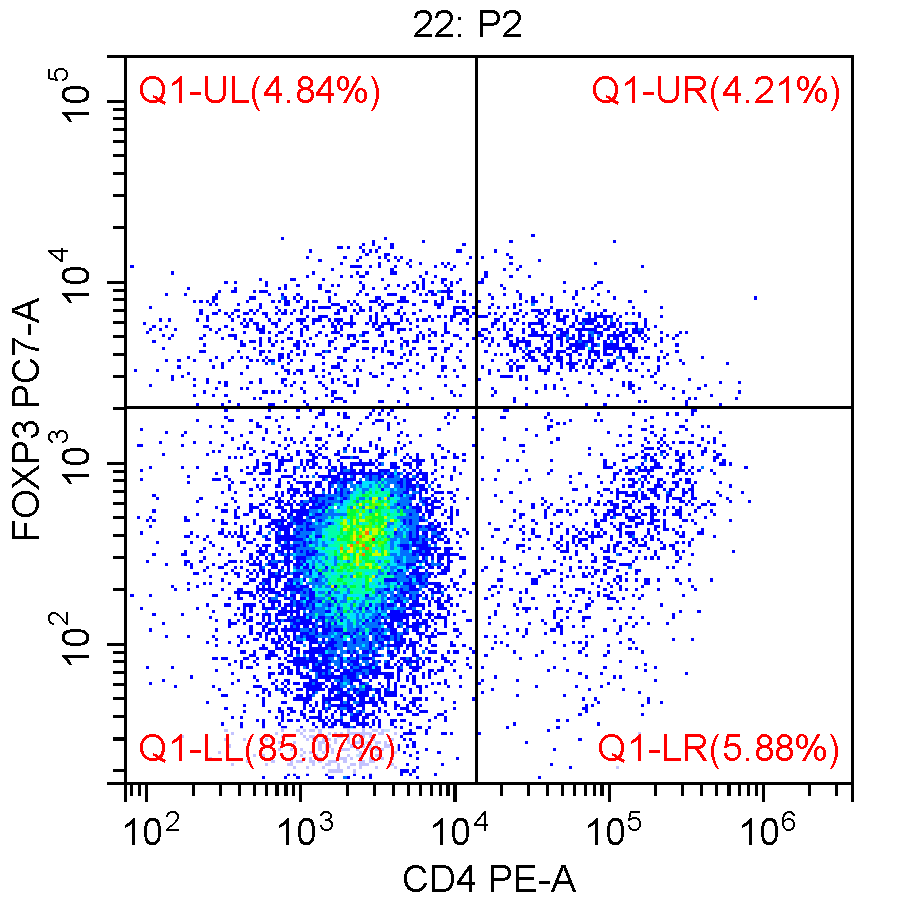

Supplement: S3 File — (ZIP) [file pone.0311419.s003.zip › ┴≈╩╜╝°╢¿+╡≥═÷/T╧╕░√▒╚╓╡/22-1.bmp]

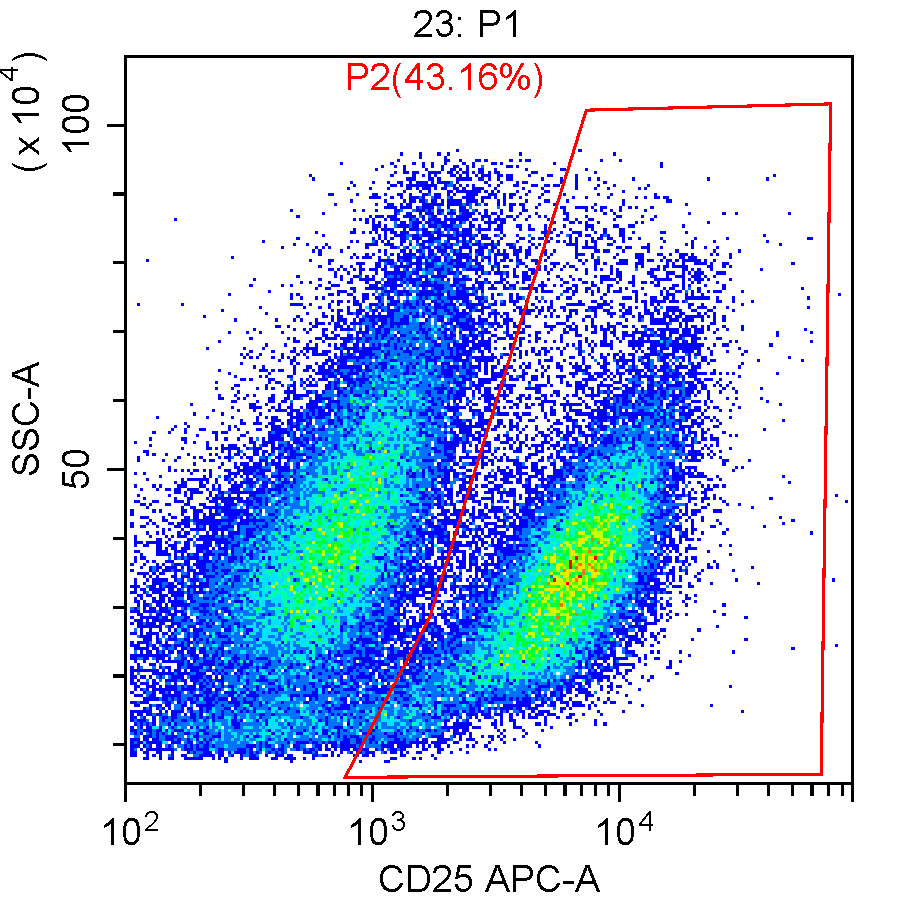

Supplement: S3 File — (ZIP) [file pone.0311419.s003.zip › ┴≈╩╜╝°╢¿+╡≥═÷/T╧╕░√▒╚╓╡/23.bmp]

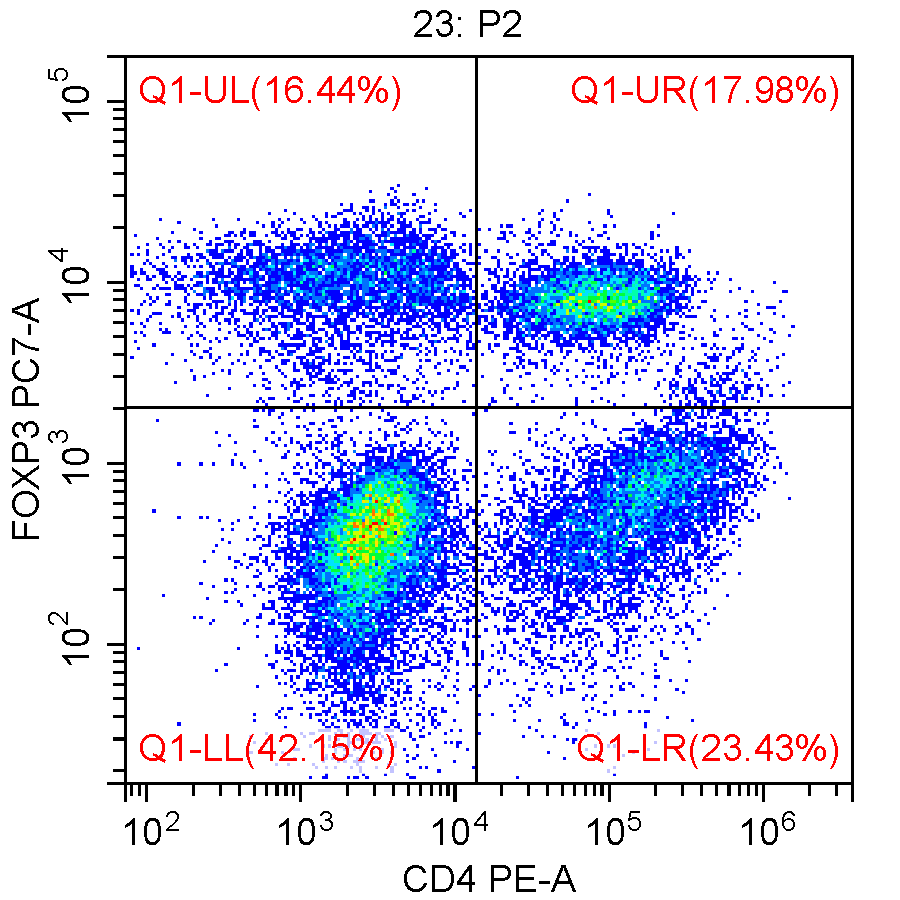

Supplement: S3 File — (ZIP) [file pone.0311419.s003.zip › ┴≈╩╜╝°╢¿+╡≥═÷/T╧╕░√▒╚╓╡/23-1.bmp]

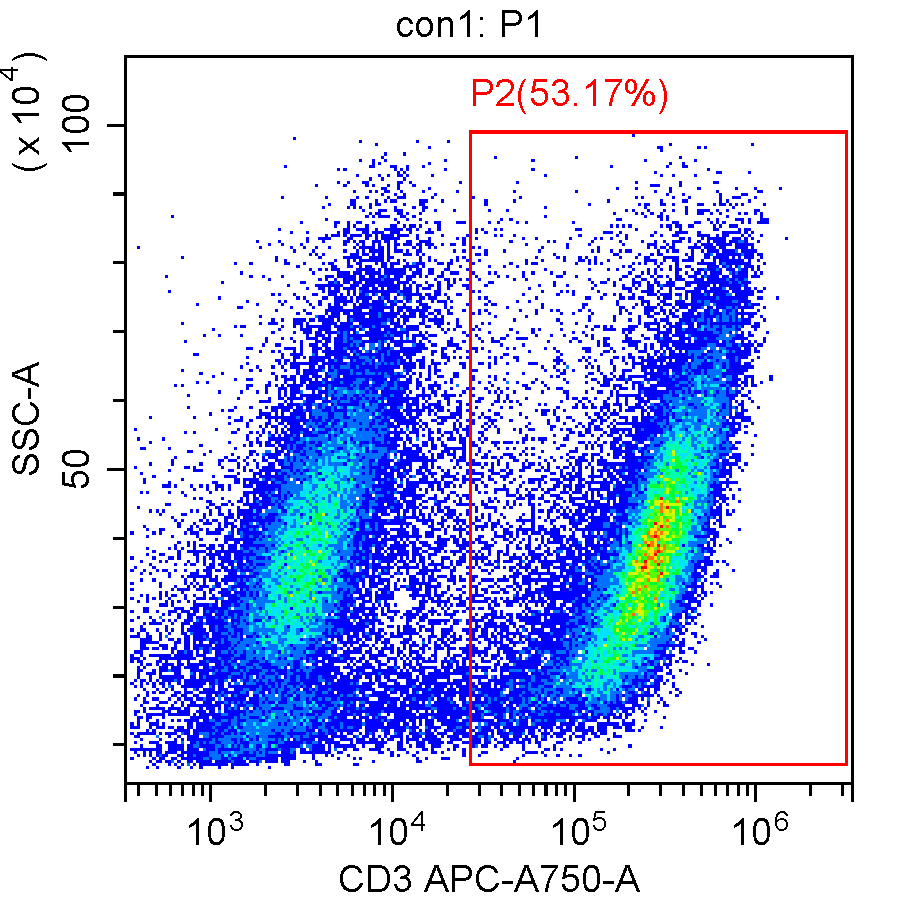

Supplement: S3 File — (ZIP) [file pone.0311419.s003.zip › ┴≈╩╜╝°╢¿+╡≥═÷/T╧╕░√▒╚╓╡/con1.bmp]

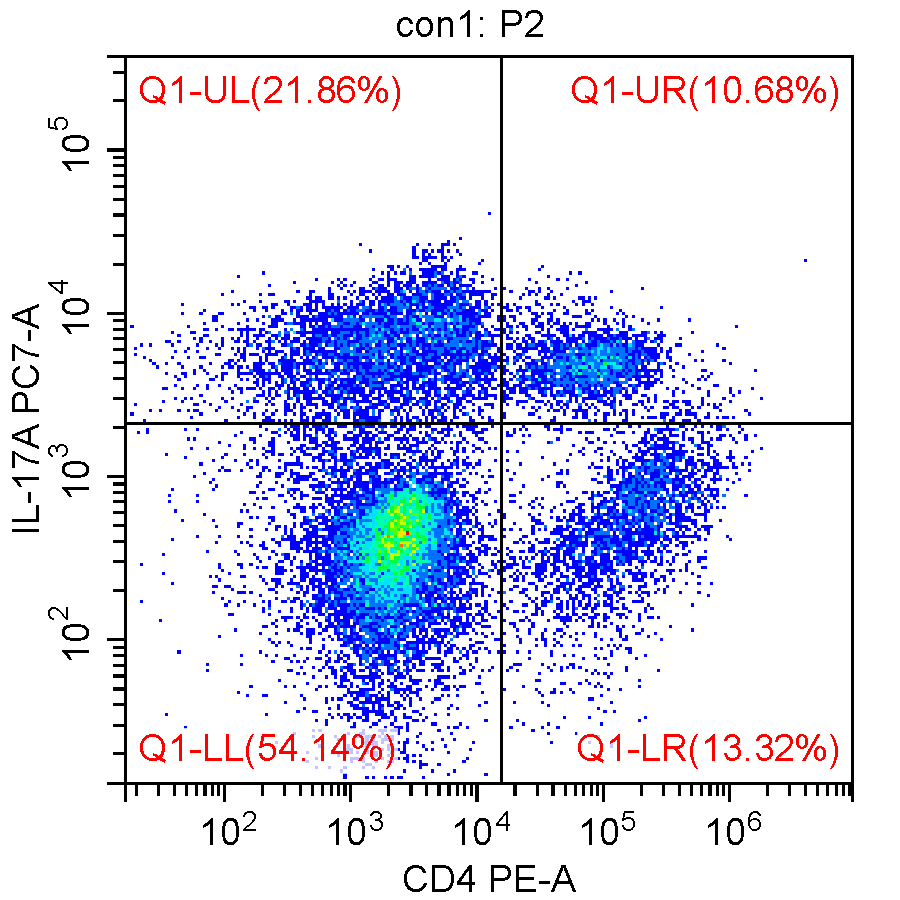

Supplement: S3 File — (ZIP) [file pone.0311419.s003.zip › ┴≈╩╜╝°╢¿+╡≥═÷/T╧╕░√▒╚╓╡/con1-1.bmp]

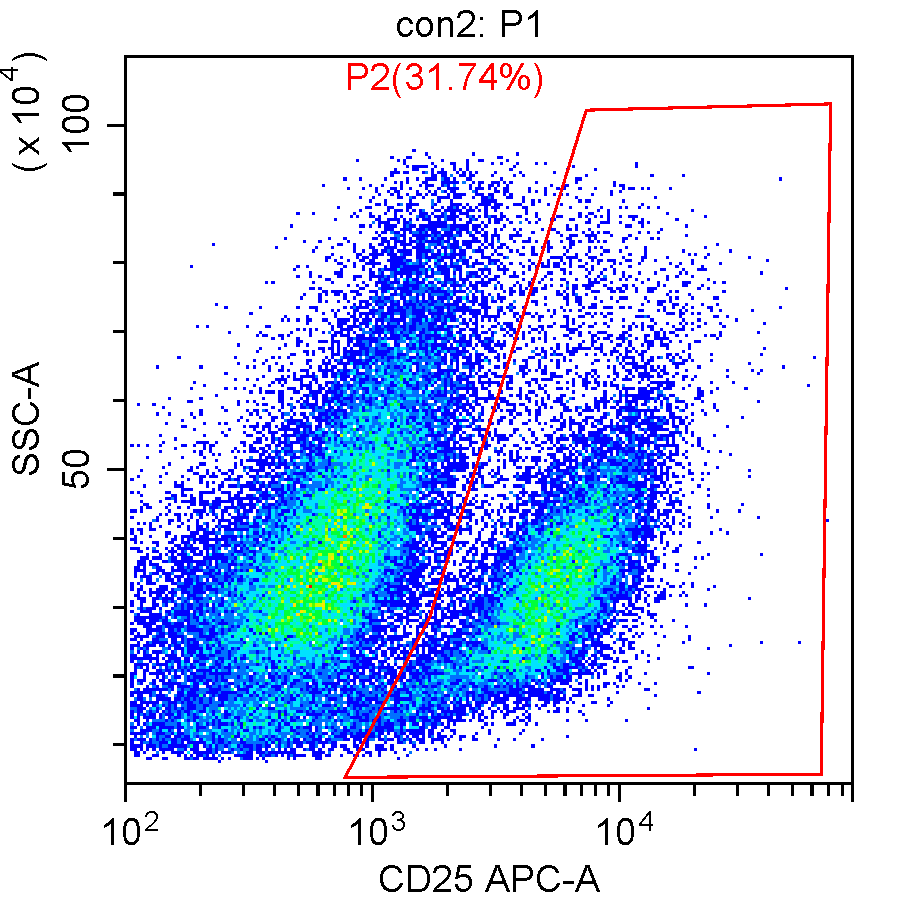

Supplement: S3 File — (ZIP) [file pone.0311419.s003.zip › ┴≈╩╜╝°╢¿+╡≥═÷/T╧╕░√▒╚╓╡/con2.bmp]

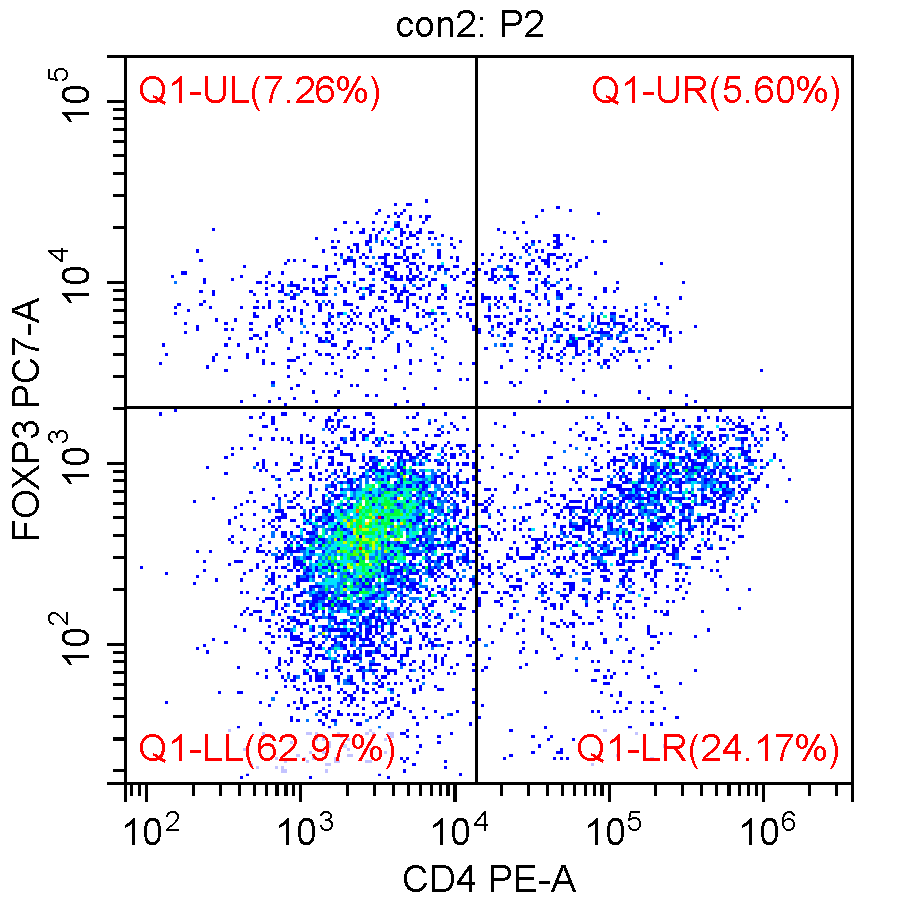

Supplement: S3 File — (ZIP) [file pone.0311419.s003.zip › ┴≈╩╜╝°╢¿+╡≥═÷/T╧╕░√▒╚╓╡/con2-1.bmp]

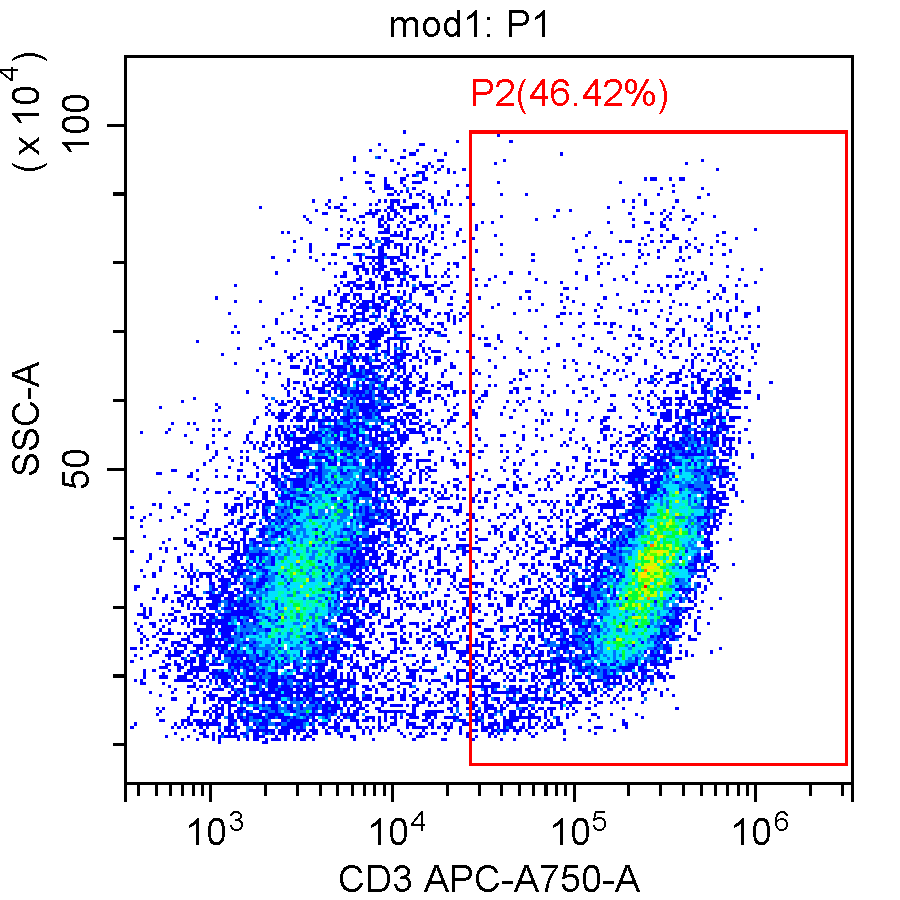

Supplement: S3 File — (ZIP) [file pone.0311419.s003.zip › ┴≈╩╜╝°╢¿+╡≥═÷/T╧╕░√▒╚╓╡/mod1.bmp]

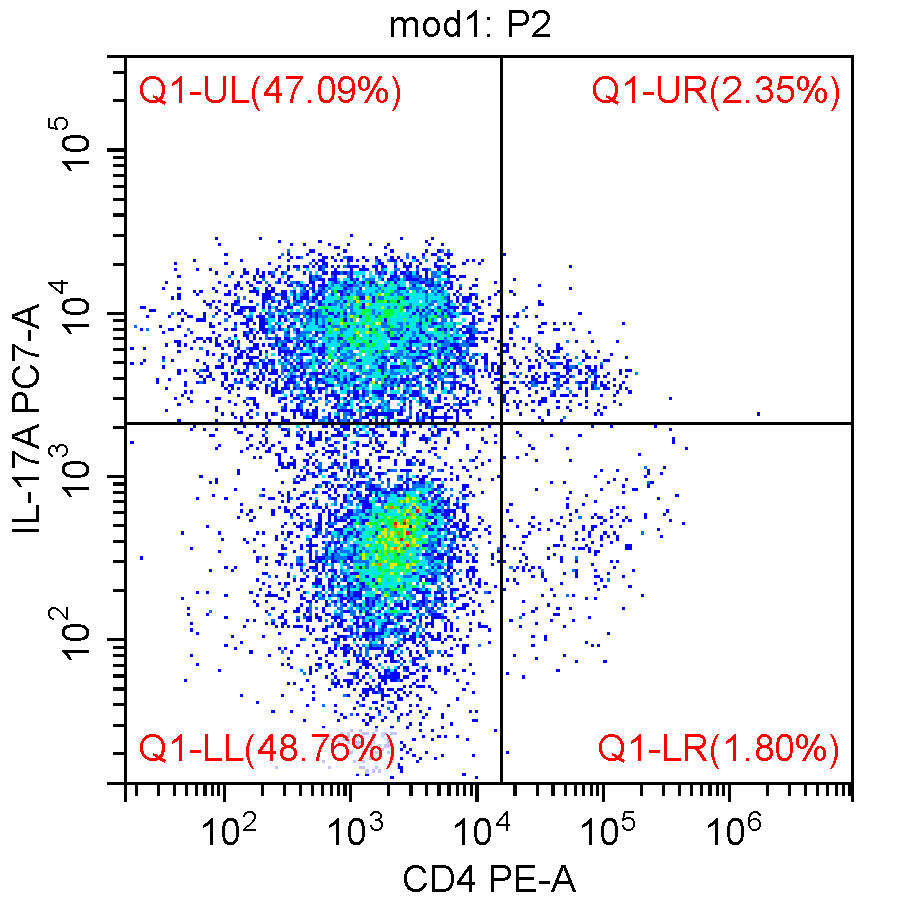

Supplement: S3 File — (ZIP) [file pone.0311419.s003.zip › ┴≈╩╜╝°╢¿+╡≥═÷/T╧╕░√▒╚╓╡/mod1-1.bmp]

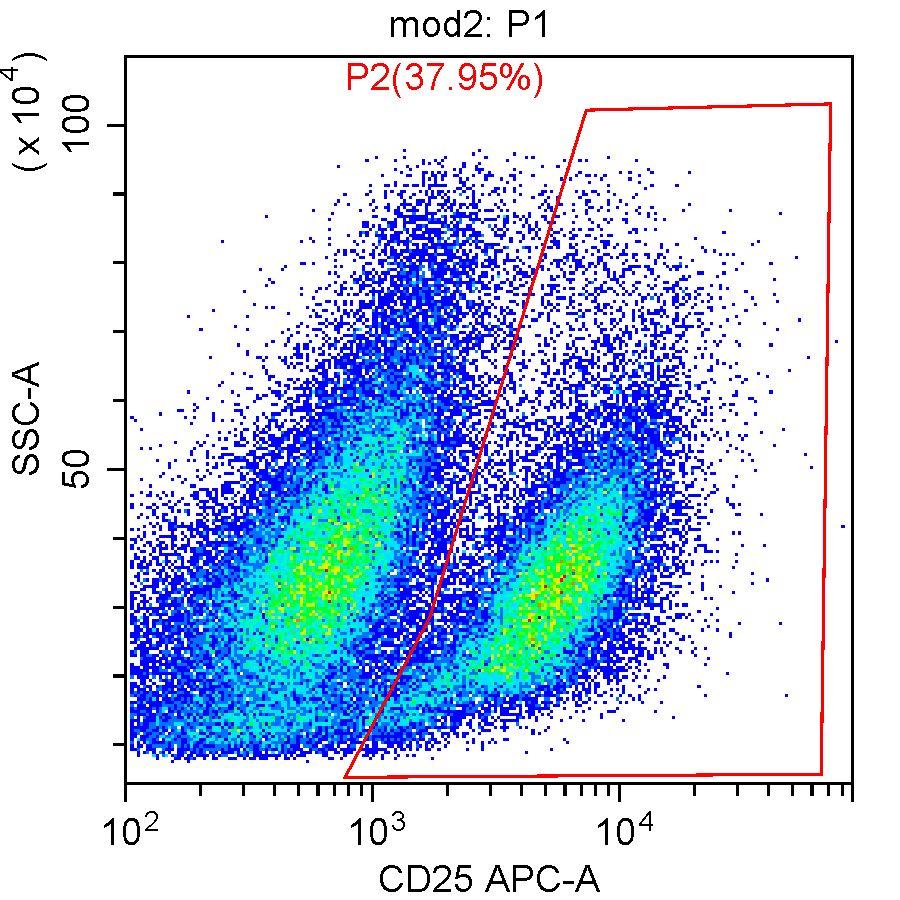

Supplement: S3 File — (ZIP) [file pone.0311419.s003.zip › ┴≈╩╜╝°╢¿+╡≥═÷/T╧╕░√▒╚╓╡/mod2.bmp]

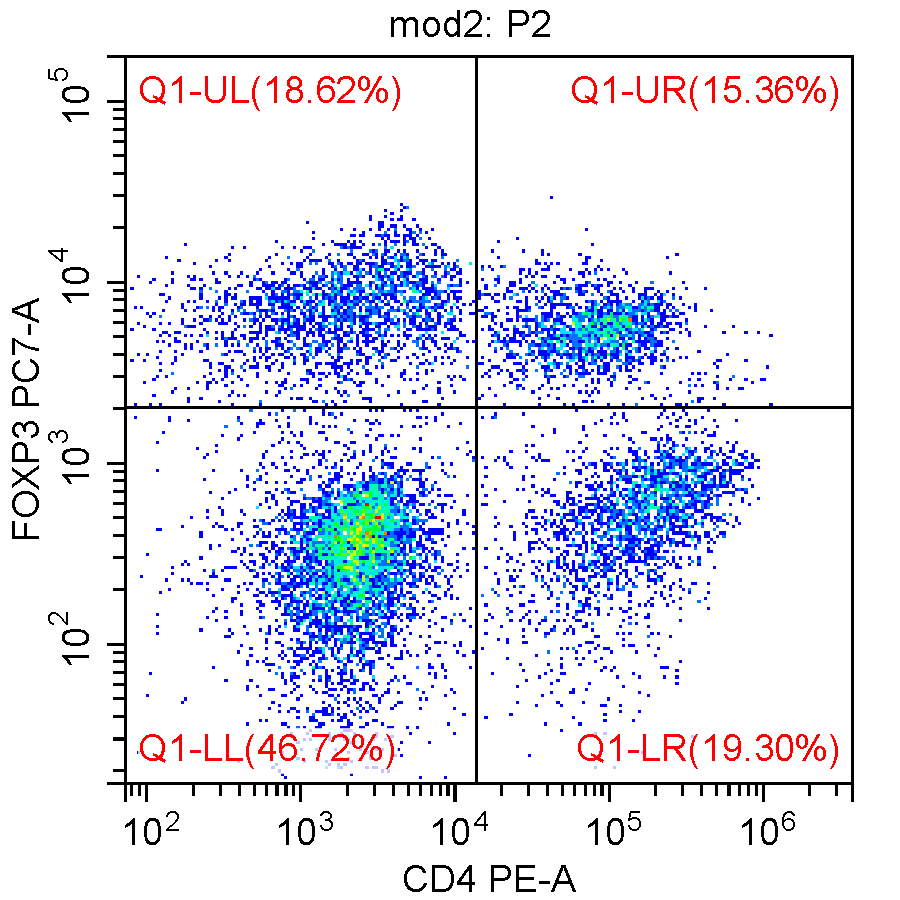

Supplement: S3 File — (ZIP) [file pone.0311419.s003.zip › ┴≈╩╜╝°╢¿+╡≥═÷/T╧╕░√▒╚╓╡/mod2-1.bmp]

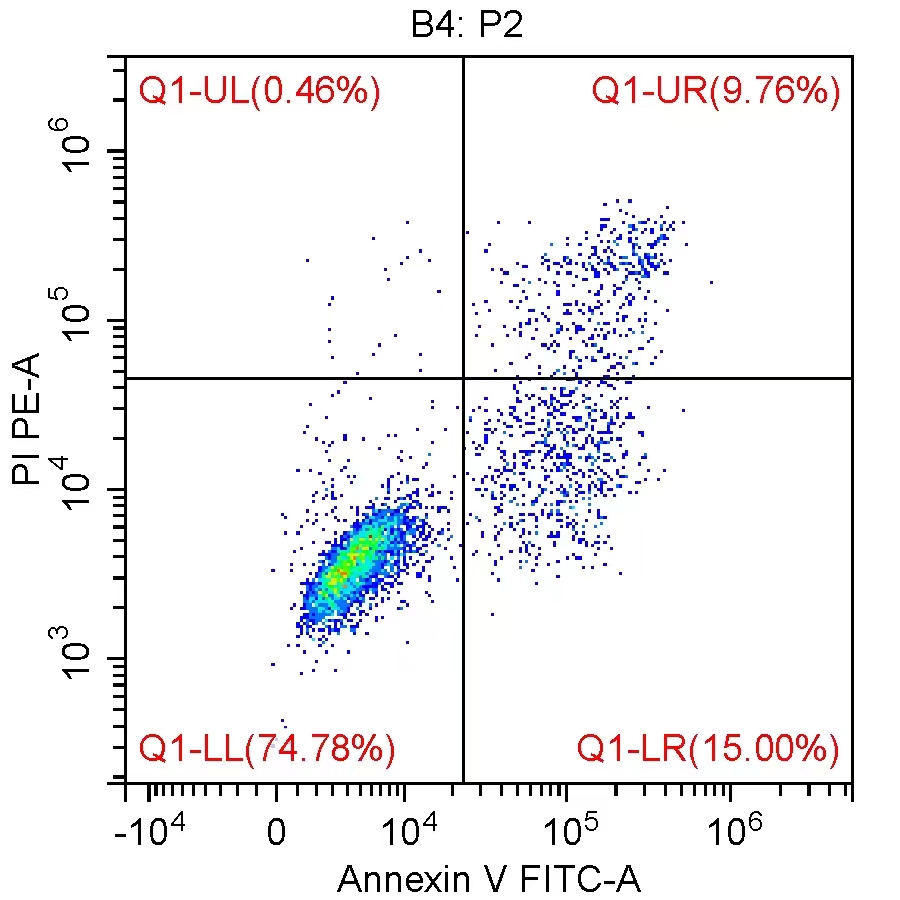

Supplement: S3 File — (ZIP) [file pone.0311419.s003.zip › ┴≈╩╜╝°╢¿+╡≥═÷/╡≥═÷/3041d394d6ad36ce4d94a98f93a0dbe.jpg]

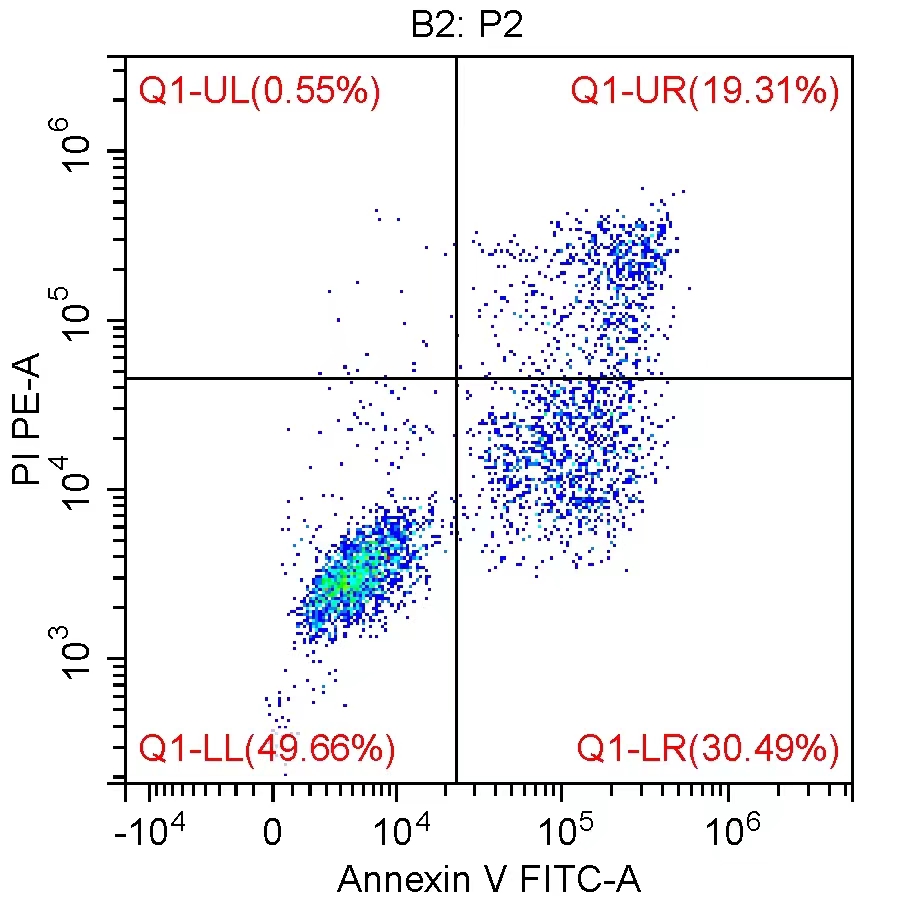

Supplement: S3 File — (ZIP) [file pone.0311419.s003.zip › ┴≈╩╜╝°╢¿+╡≥═÷/╡≥═÷/6f30785b2464e28332659d4bed04edc.jpg]

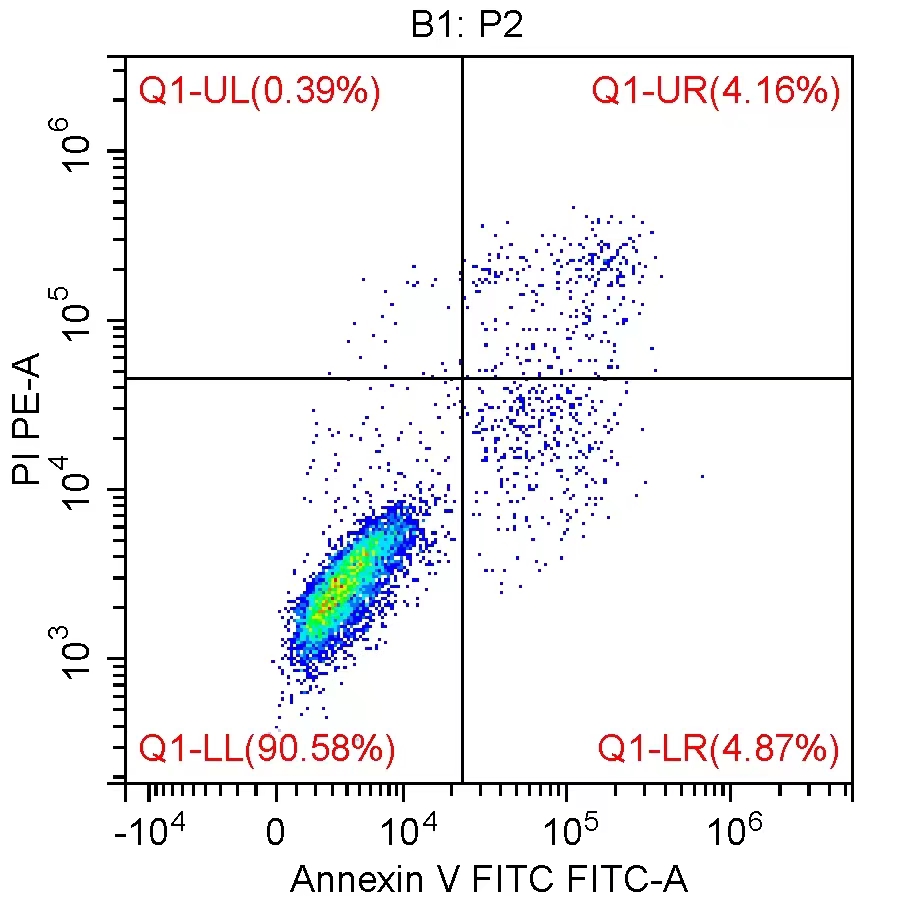

Supplement: S3 File — (ZIP) [file pone.0311419.s003.zip › ┴≈╩╜╝°╢¿+╡≥═÷/╡≥═÷/b32b0971466e2f409e0492a4f1aff6b.jpg]

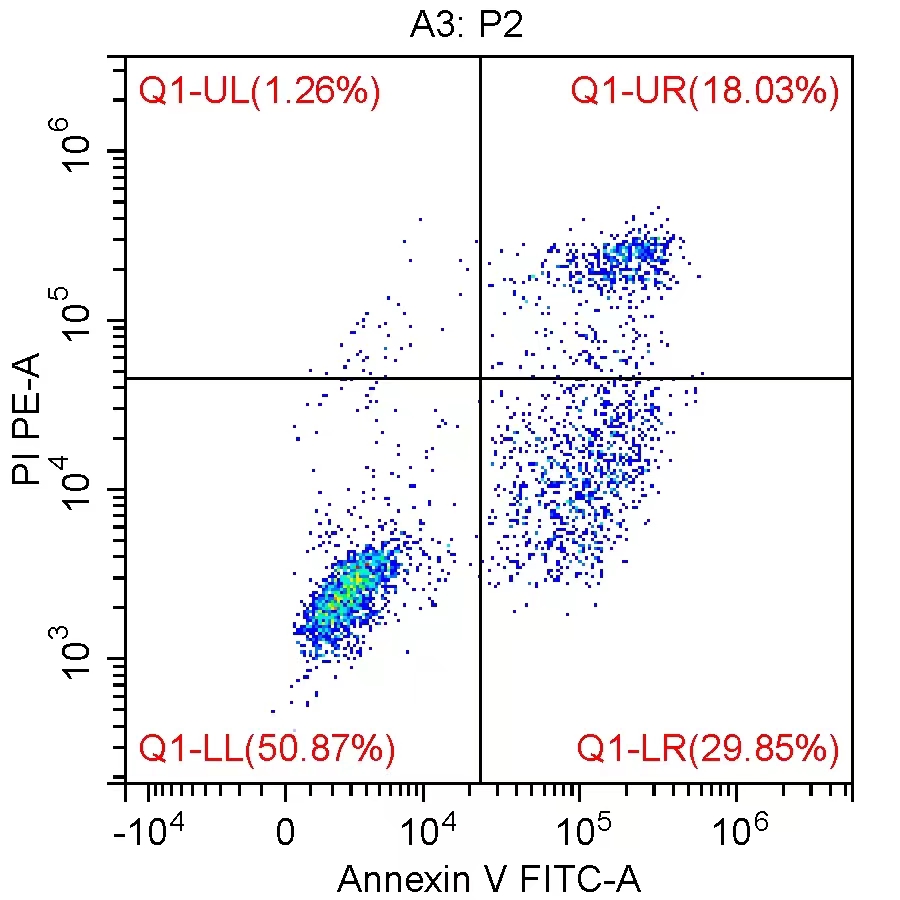

Supplement: S3 File — (ZIP) [file pone.0311419.s003.zip › ┴≈╩╜╝°╢¿+╡≥═÷/╡≥═÷/b4d4c612a6be25e1bdc8574fd94fdda.jpg]

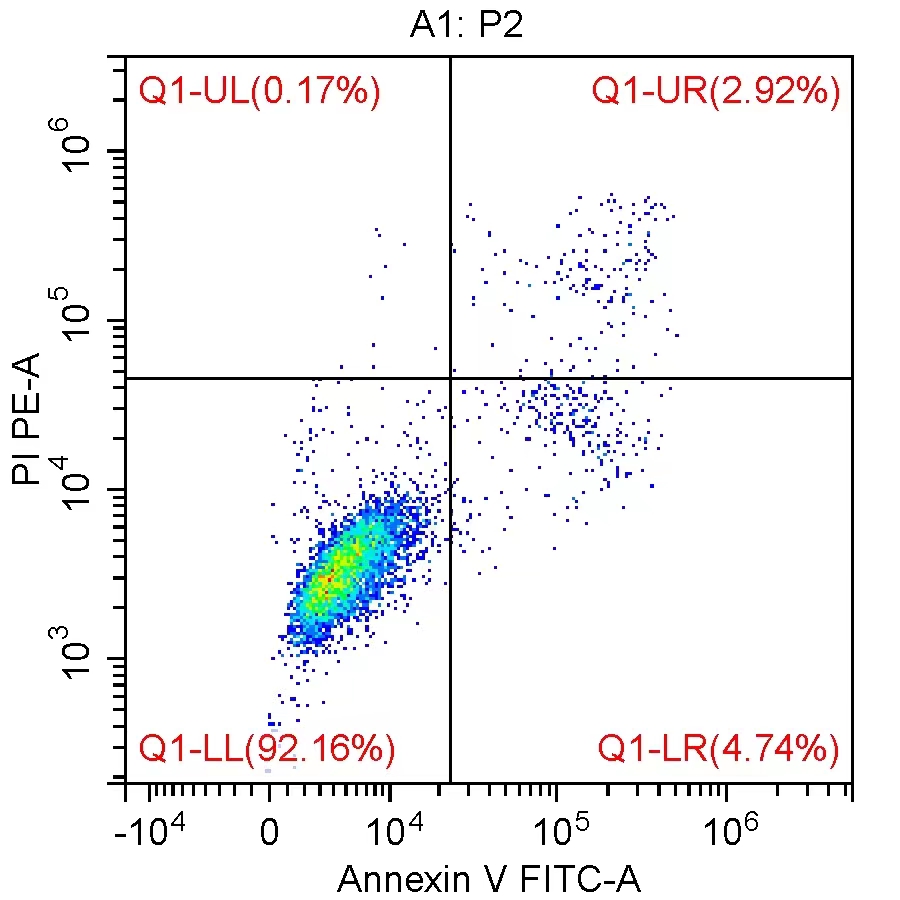

Supplement: S3 File — (ZIP) [file pone.0311419.s003.zip › ┴≈╩╜╝°╢¿+╡≥═÷/╡≥═÷/bfd26ea7c991e66be9c0884f2e5b542.jpg]

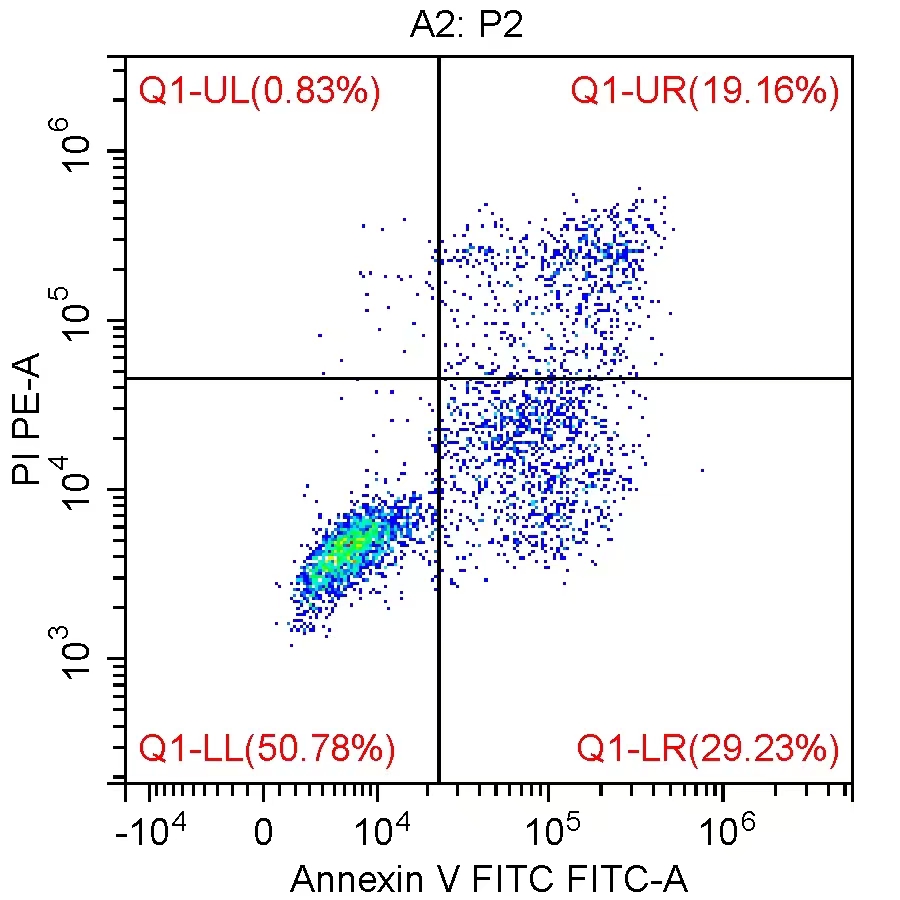

Supplement: S3 File — (ZIP) [file pone.0311419.s003.zip › ┴≈╩╜╝°╢¿+╡≥═÷/╡≥═÷/ccd3a09395f1fd6fcb5683f610e6b58.jpg]

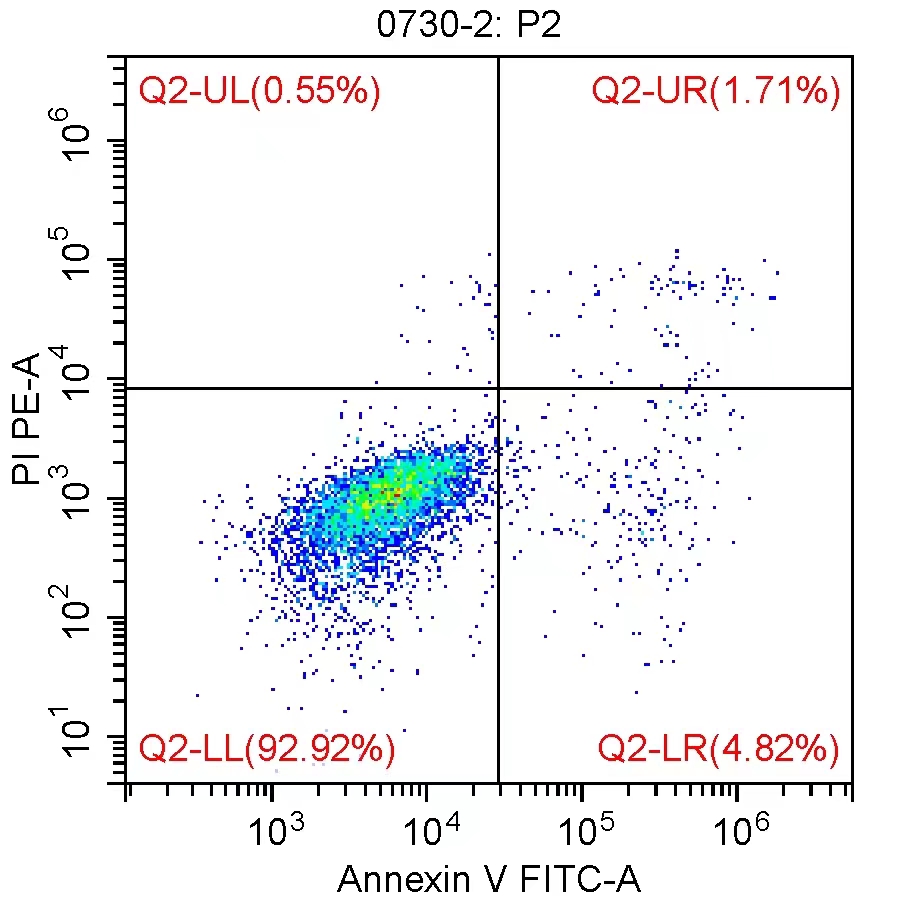

Supplement: S3 File — (ZIP) [file pone.0311419.s003.zip › ┴≈╩╜╝°╢¿+╡≥═÷/╡≥═÷/e00d51d72c5e440e2a589b3891dabc8.jpg]

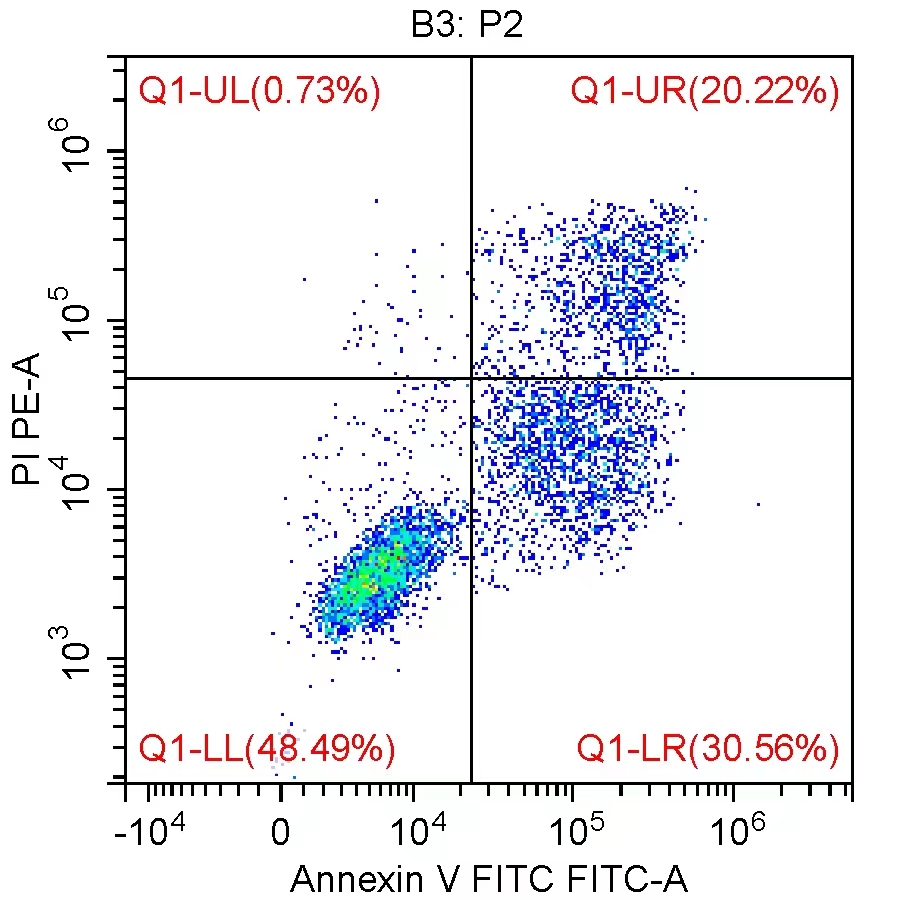

Supplement: S3 File — (ZIP) [file pone.0311419.s003.zip › ┴≈╩╜╝°╢¿+╡≥═÷/╡≥═÷/f39e653ad2062d24b2df14aa1a69fed.jpg]
