# Supplementary material for: MiR-27a inhibits the growth and metastasis of multiple myeloma through regulating Th17/Treg balance
Source: PLoS One. 2024 Oct 16;19(10):e0311419. doi: 10.1371/journal.pone.0311419 (PMC11482689; doi:10.1371/journal.pone.0311419)
Supplement: Attachment — Submitted filename: Responses.docx [file pone.0311419.s005.docx]

**Dear editor and reviewers,**

Thank you for your thorough review and valuable feedback. We have diligently addressed the identified deficiencies in our manuscript.

All concerns raised by the reviewer have been meticulously addressed, and we have made revisions to both figures and text accordingly.

Specifically, we have uploaded our original research data to the Harvard Dataverse. The dataset includes experimental results, statistical analyses, and any additional data required to replicate the study’s findings in their entirety. All datasets have been anonymized to remove any potentially identifying patient information. The data can be accessed at the following link: <https://doi.org/10.7910/DVN/BZATPL>.

All modified sections are clearly marked in red font in the revised manuscript.

We believe these revisions have significantly improved the clarity and completeness of our manuscript.

Thank you for your guidance, and we remain open to further suggestions or clarifications.

Best regards

Mingfeng Xiao

[xiaomingfeng2023@163.com](mailto:xiaomingfeng2023@163.com)

**Below is a point-by-point response to the editor's comments:**

1. Please ensure that your manuscript meets PLOS ONE's style requirements, including those for file naming. The PLOS ONE style templates can be found at

https://journals.plos.org/plosone/s/file?id=wjVg/PLOSOne_formatting_sample_main_body.pdf andhttps://journals.plos.org/plosone/s/file?id=ba62/PLOSOne_formatting_sample_title_authors_affiliations.pdf

Thank you for bringing this to our attention. We have reviewed the PLOS ONE style requirements and have made the necessary corrections to ensure that our manuscript meets these standards, including proper file naming.

2. To comply with PLOS ONE submissions requirements, in your Methods section, please provide additional information regarding the experiments involving animals and ensure you have included details on (1) methods of anesthesia and/or analgesia, and (2) efforts to alleviate suffering

Thank you for your feedback. We have updated the Methods section to include additional details on the experiments involving animals. We used 1.5% isoflurane for inhalation anesthesia. Efforts to alleviate suffering included monitoring animals for signs of distress and providing appropriate analgesia when necessary.

3. We noticed you have some minor occurrence of overlapping text with the following previous publication(s), which needs to be addressed: https://doi.org/10.1007/s10753-014-9980-4 In your revision ensure you cite all your sources (including your own works), and quote or rephrase any duplicated text outside the methods section. Further consideration is dependent on these concerns being addressed.

Thank you for bringing this to our attention. We have carefully reviewed the mentioned publication and confirmed that our study investigates a completely different mechanism. To address the concern, we have revised our manuscript to avoid any overlapping text and have properly cited the referenced areas where appropriate.

4. Thank you for stating the following financial disclosure: "1.Administration of Traditional Chinese Medicine of Guangdong Province, China，Fund No.20241093；2.Foundation of Guangdong Province, China，Fund No.C2023074" Please state what role the funders took in the study. If the funders had no role, please state: "The funders had no role in study design, data collection and analysis, decision to publish, or preparation of the manuscript." If this statement is not correct you must amend it as needed. Please include this amended Role of Funder statement in your cover letter; we will change the online submission form on your behalf.

Thank you for your feedback. Thank you for your feedback. The recipients of the funding were responsible for the conception and design of this study.

5. In your Data Availability statement, you have not specified where the minimal data set underlying the results described in your manuscript can be found. PLOS defines a study's minimal data set as the underlying data used to reach the conclusions drawn in the manuscript and any additional data required to replicate the reported study findings in their entirety. All PLOS journals require that the minimal data set be made fully available. For more information about our data policy, please see <http://journals.plos.org/plosone/s/data-availability>. Upon re-submitting your revised manuscript, please upload your study’s minimal underlying data set as either Supporting Information files or to a stable, public repository and include the relevant URLs, DOIs, or accession numbers within your revised cover letter. For a list of acceptable repositories, please see http://journals.plos.org/plosone/s/data-availability#loc-recommended-repositories. Any potentially identifying patient information must be fully anonymized. Important: If there are ethical or legal restrictions to sharing your data publicly, please explain these restrictions in detail. Please see our guidelines for more information on what we consider unacceptable restrictions to publicly sharing data: http://journals.plos.org/plosone/s/data-availability#loc-unacceptable-data-access-restrictions. Note that it is not acceptable for the authors to be the sole named individuals responsible for ensuring data access. We will update your Data Availability statement to reflect the information you provide in your cover letter.

Thank you for your feedback. We have uploaded our original research data to the Harvard Dataverse. The dataset includes experimental results, statistical analyses, and any additional data required to replicate the study’s findings in their entirety. All datasets have been anonymized to remove any potentially identifying patient information. The data can be accessed at the following link: <https://doi.org/10.7910/DVN/BZATPL>.

6. PLOS ONE now requires that authors provide the original uncropped and unadjusted images underlying all blot or gel results reported in a submission’s figures or Supporting Information files. This policy and the journal’s other requirements for blot/gel reporting and figure preparation are described in detail at https://journals.plos.org/plosone/s/figures#loc-blot-and-gel-reporting-requirements and https://journals.plos.org/plosone/s/figures#loc-preparing-figures-from-image-files. When you submit your revised manuscript, please ensure that your figures adhere fully to these guidelines and provide the original underlying images for all blot or gel data reported in your submission. See the following link for instructions on providing the original image data: https://journals.plos.org/plosone/s/figures#loc-original-images-for-blots-and-gels.

In your cover letter, please note whether your blot/gel image data are in Supporting Information or posted at a public data repository, provide the repository URL if relevant, and provide specific details as to which raw blot/gel images, if any, are not available. Email us at plosone@plos.org if you have any questions.

We have prepared and uploaded the original uncropped and unadjusted images underlying all blot and gel results as required. These images have been provided in the Supporting Information files associated with our revised manuscript.

7. Please include captions for your Supporting Information files at the end of your manuscript, and update any in-text citations to match accordingly. Please see our Supporting Information guidelines for more information: http://journals.plos.org/plosone/s/supporting-information.

Thank you for your guidance. We have now included captions for all Supporting Information files at the end of our manuscript, and we have updated the in-text citations accordingly to match these captions.

**Below is a point-by-point response to the reviewer's comments:**

**Responses to Reviewer #1**

1. Text on line 121 has a different font color.

Thank you for your feedback. The font color on line 121 has been corrected.

2. Was DNA purity tested by running a gel too?

Thank you for your feedback. In this experiment, we did test DNA purity by running a gel as part of our routine pre-experiment procedures.

3. Where were primers purchased from?

Thank you for your feedback. I have now included the information about where the primers were purchased in the manuscript.

4. Were cells serum-starved prior to viability and apoptosis assays?

Thank you for your insightful question. Yes, the cells were serum-starved prior to the viability and apoptosis assays. This step is essential for studying cell viability and apoptosis. We have now included this information in the manuscript, specifying that the cells were cultured in 0.5% serum for 12 hours before the experiments.

5. GEO database analysis lacks details of how analysis was conducted, there is not sufficient detail for the study to be replicated.

We have addressed the concerns raised in your review in our revised submission. We have provided a comprehensive explanation of our data normalization and differential expression analysis methods in the Methods section.

6. Just to clarify, only 3 normal and 3 MM datasets were selected? This is what is represented in the clusterogram.

Indeed, there are 19 patients included in the study, which means 19 patients with 3 MM and 3 paired normal tissues. were used for the analysis, as represented in the clusterogram.

7. Figure 2B, perhaps include MiR-27a in the y axis label.

Figure 2B specifically depicts the expression levels of MiR-27a. We have provided clear clarification in the figure legend below.

8. Figure 3C legend does not fully describe what is seen. This is also not well described in text.

Thank you for your feedback. We have now provided a more detailed description of Figure 3C in both the figure legend and the text.

9. I am not fully convinced by this statement: initial validation of the regulatory impact of the MiR-27a/PI3K/AKT/mTOR axis on the modulation of Th17/Treg balance in MM. The study does show the dual functionality of MiR-27A but does not clearly validate the regulatory impact of the MiR-27a/PI3K/AKT/mTOR axis on the modulation of Th17/Treg balance in MM. Although this study showed that miR-27a impacts the development of MM through the PI3K/AKT/mTOR signaling pathway, this cannot infer its modulation of the Th17/Treg balance. This is a major concern as it is the basis of the study and a conclusion that is drawn. The study does hold significant merit in its findings regarding the PI3K/AKT/mTOR pathway and MiR-27a.

We appreciate your insightful feedback and agree with your suggestion. Indeed, our study includes a rescue experiment in cell assays, demonstrating the significant impact of MiR-27a on the PI3K/AKT/mTOR pathway. However, we acknowledge the limitations in our experimental design, which only provide preliminary findings. To address your concerns comprehensively, additional experiments, such as genetic knockout animal models, would be necessary. These experiments are essential for fully validating our hypothesis. We are committed to addressing these aspects in future research endeavors to enhance the robustness of our conclusions.

**Responses to Reviewer #2**

1. In the abstract the conclusion is presented very vaguely, please improve the sentence structure to make it more scientific and clear to understand.

Thank you for your valuable feedback. We have revised the conclusion in the abstract to improve its scientific clarity and readability.

2. Introduction is satisfactory, the last lines on page 4 (84to 87) needs clarity of the aim of studies.

Thank you for your feedback. We have revised the last lines to provide greater clarity regarding the aim of our studies.

3. Methods are presented well, however certain details are missing. Please explain what is meant by control in case of cells are they untransfected cells or mock transfected? The information about the designing of miR-27a mimic and anti-miR-27a is missing. How the mutant of PI3K was constructed? Experiments replication number is not mentioned. The ethical permissions for animal studies should also be mentioned in methods section.

Thank you for your detailed feedback. We have revised the methods section to address the missing details. The control group consists of mock transfected cells, serving as a negative control. The experiments were replicated three times. We have also included the ethical permissions for animal studies as required. Regarding the miR-27a mimic and anti-miR-27a, we designed the mimic to match the mature miR-27a sequence and the anti-miR-27a as a complementary sequence to inhibit miR-27a function MiR-27a mimic and anti-miR-27a were obtained from GenePharma Co. Ltd. (Shanghai, China). The PI3K activator 740 Y-P and its inhibitor, 3-Methyladenine, were provided by MedChemExpress (Shanghai, China).

4. In the Results the statistical analysis in all the figures is not clear, the authors should double check all the analysis as at some points e.g in fig 2 it says **** but in the figure caption it is not mentioned. Also in the mRNA analysis the values on y-axis and stats doesnot coordinate. Please double check.

Figure 3c shows unequal contrast of the images. Fig 4 is not visible in the document. The authors may show some other in-vitro metastasis assays such as ECM degradation to support their hypothesis with appropriate controls.

Thank you for your detailed feedback. We have updated the manuscript and figures to include the relevant statistical analysis, ensuring clarity and consistency throughout. We have also corrected the y-axis values and statistics in the mRNA analysis to ensure proper coordination. Figure 4 is now visible in the document. While we acknowledge the importance of ECM degradation assays, we believe that our transwell assays and flow cytometry apoptosis assays sufficiently reflect tumor phenotypes. Our primary focus is on the impact of multiple myeloma on the Treg/Th17 cell balance, which is the main objective of our study. Additionally, to strengthen our conclusions, we have included an additional cell line for validation.

5.The discussion section needs improvement in corelating the results to the conclusion made. The authors have provided enough references from the other studies but failed to corelate their own results with the conclusions made. Please write few sentences in this regard.

Thank you for your constructive feedback. We have revised the discussion section to better correlate our results with the conclusions made, providing a clearer connection between our findings and the study's implications.

6. Overall the sentence structure could be improved throughout the document.

Thank you for your valuable feedback. We have engaged a professional language editing service to improve the sentence structure throughout the document.
