# Supplementary material for: MiR-27a inhibits the growth and metastasis of multiple myeloma through regulating Th17/Treg balance
Source: PLoS One. 2024 Oct 16;19(10):e0311419. doi: 10.1371/journal.pone.0311419 (PMC11482689; doi:10.1371/journal.pone.0311419)
Supplement: Attachment — Submitted filename: Responses.docx [file pone.0311419.s006.docx]

**Dear editor and reviewers,**

Thank you for your thorough review and valuable feedback. We have diligently addressed the identified deficiencies in our manuscript.

All concerns raised by the reviewer have been meticulously addressed. All modified sections are clearly marked in red font in the revised manuscript.

We believe these revisions have significantly improved the clarity and completeness of our manuscript.

Thank you for your guidance, and we remain open to further suggestions or clarifications.

Best regards

Mingfeng Xiao

[xiaomingfeng2023@163.com](mailto:xiaomingfeng2023@163.com)

**Below is a point-by-point response to the reviewer's comments:**

**Responses to Reviewer #2**

1. There should be consistency in writing miR-27a. At some places it's written with 'M" and at others with "m".

Thank you for your feedback. We have revised the manuscript to ensure consistency in writing miR-27a. All instances of "M" have been changed to "m" except at the beginning of sentences.

2. Please look for sentence structure errors.

Thank you for your suggestion. We have had the manuscript professionally reviewed and edited by native English speaker to address any sentence structure errors.
